# Analysis of whole genome-transcriptomic organization in brain to identify genes associated with alcoholism

**DOI:** 10.1038/s41398-019-0384-y

**Published:** 2019-02-14

**Authors:** Manav Kapoor, Jen-Chyong Wang, Sean P. Farris, Yunlong Liu, Jeanette McClintick, Ishaan Gupta, Jacquelyn L. Meyers, Sarah Bertelsen, Michael Chao, John Nurnberger, Jay Tischfield, Oscar Harari, Li Zeran, Victor Hesselbrock, Lance Bauer, Towfique Raj, Bernice Porjesz, Arpana Agrawal, Tatiana Foroud, Howard J. Edenberg, R. Dayne Mayfield, Alison Goate

**Affiliations:** 1 Department of Neuroscience, Icahn School of Medicine at Mt. Sinai, 1425 Madison Ave, New York, NY USA; 2 The Waggoner Center for Alcohol and Addiction Research, The University of Texas at Austin, Austin, TX 78712 USA; 3 Department of Medical and Molecular Genetics, Indiana University School of Medicine, Indianapolis, IN USA; 4 Department of Biological Sciences, Indian Institute of Science Education and Research, Bhopal, MP 462066 India; 5 Department of Psychiatry, Henri Begleiter Neurodynamics Lab, State University of New York, Downstate Medical Center, Brooklyn, NY USA; 6 Department of Genetics and the Human Genetics Institute of New Jersey, Rutgers University, Piscataway, NJ USA; 7 Department of Psychiatry, Washington University School of Medicine in St. Louis, St. Louis, MO USA; 8 Department of Psychiatry, University of Connecticut, Farmington, CT USA

## Abstract

Alcohol exposure triggers changes in gene expression and biological pathways in human brain. We explored alterations in gene expression in the Pre-Frontal Cortex (PFC) of 65 alcoholics and 73 controls of European descent, and identified 129 genes that showed altered expression (FDR < 0.05) in subjects with alcohol dependence. Differentially expressed genes were enriched for pathways related to interferon signaling and Growth Arrest and DNA Damage-inducible 45 (GADD45) signaling. A coexpression module (thistle2) identified by weighted gene co-expression network analysis (WGCNA) was significantly correlated with alcohol dependence, alcohol consumption, and AUDIT scores. Genes in the thistle2 module were enriched with genes related to calcium signaling pathways and showed significant downregulation of these pathways, as well as enrichment for biological processes related to nicotine response and opioid signaling. A second module (brown4) showed significant upregulation of pathways related to immune signaling. Expression quantitative trait loci (eQTLs) for genes in the brown4 module were also enriched for genetic associations with alcohol dependence and alcohol consumption in large genome-wide studies included in the Psychiatric Genetic Consortium and the UK Biobank’s alcohol consumption dataset. By leveraging multi-omics data, this transcriptome analysis has identified genes and biological pathways that could provide insight for identifying therapeutic targets for alcohol dependence.

## Introduction

Alcohol dependence (AD) can be defined as a cluster of physiological, behavioral, and cognitive phenomena in which the use of alcohol takes a much higher priority for a given individual than other behaviors that once had greater value (American Psychiatric Association 1994)^1^. The development of AD is characterized by frequent episodes of intoxication, preoccupation with alcohol, use of alcohol despite adverse consequences, compulsion to seek and consume alcohol, loss of control in limiting alcohol intake, and emergence of a negative emotional state in the absence of the drug (American Psychiatric Association 1994)^1^. The changes in behavioral priorities not only results in increased morbidity and mortality, it is also a substantial social and economic burden on individual families and the nation^2^.

In individuals with alcohol dependence, there is a complex interplay between genetic background, environmental factors, and history of alcohol exposure^3^. Alcohol crosses the blood brain barrier and triggers changes in the central nervous system^4^, including transcriptional changes in many different regions of the brain^5–9^. The transcriptional effects of long-term alcohol consumption are associated with pathways involved in the neuro-immune system, neurotoxicity, and changes in neuroplasticity^6,7,9^. Transcriptomes from complex tissues, such as human brain, may be organized into networks of co-expressed genes that better reflect the biological functions and organization of the tissue^7–14^. Application of bioinformatics techniques, such as weighted gene co-expression network analysis (WGCNA)^15^, has uncovered networks associated with alcohol dependence^8,9^. However, past studies were performed on small numbers of AD cases, thus limiting the statistical power to detect small changes in alcohol-induced gene expression. In this study, we utilized massively parallel sequencing of RNA transcripts from postmortem human prefrontal cortex (PFC) of 65 alcoholics and 73 controls of European descent to explore transcriptional networks and genetic variation and identified groups of coexpressed genes associated with alcohol dependence. Our analysis provides systems-level evidence of genetic networks within the PFC that contribute to the pathophysiology of alcohol drinking behavior in humans.

## Materials and methods

### Case selection and postmortem tissue collection

Human autopsy brain samples were obtained from the New South Wales Tissue Resource Centre at the University of Sydney (http://sydney.edu.au/medicine/pathology/btrc/). Fresh frozen samples of the superior frontal gyrus (Brodmann area 8; referred to as prefrontal cortex (PFC) in this manuscript) were collected from each postmortem sample. Brain tissue was sectioned at 3 mm intervals in the coronal plane. Alcohol-dependent diagnoses were confirmed by physician interviews, review of hospital medical records, questionnaires to next-of-kin, and from pathology, radiology, and neuropsychology reports. Tissue samples were matched as closely as possible according to age, sex, post-mortem interval, pH of tissue, disease classification, and cause of death. To be included as part of the alcohol-dependent cohort, subjects had to meet the following criteria: greater than 18 years of age, no head injury at time of death, lack of developmental disorder, no recent cerebral stroke, no history of other psychiatric or neurological disorders, no history of intravenous or polydrug abuse, negative screen for AIDS and hepatitis B/C, post-mortem interval within 48 h, and diagnosis of AD meeting the DSM-IV criteria^1^.

### Sample preparation

The Qiagen RNeasy and Lipid Tissue kit (Qiagen, Valencia, CA, USA) was used to extract total RNA from human PFC brain tissue, and RNA concentration was measured with a NanoDrop 8000 spectrophotometer (ThermoFisher Scientific). An Agilent Bioanalyzer (Agilent Technologies, Santa Clara, CA, USA) was used to test the integrity of RNA samples. Samples with an RNA integrity number (RIN) < 5.5 were removed from futher analyses. Sixty samples were processed at the Waggoner Center for Alcohol and Addiction Research (WCAAR), The University of Texas at Austin while 83 samples were processed at the Ronald M. Loeb Center for Alzheimer disease, Icahn School of Medicine at Mount Sinai. Details about the library preparation and sequencing is provided in the supplementary document.

### Mapping and quantification of gene expression

Raw reads were aligned to human genome 19 (hg19) using STAR aligner (version 2.5.3.a)^16^. We used QC tools RSeQC (http://code.google.com/p/rseqc/) and Picard (https://broadinstitute.github.io/picard/) to evaluate RNA sequence quality including the %GC, %duplicates, gene body coverage, unsupervised clustering, and the library complexity. We used the Picard “MarkDuplicates” option to flag and remove duplicate reads. Gene quantification was performed with featureCounts (SUBREAD package; release 1.6.0)^17^ using Gencode annotations (Release 19 (GRCh37.p13)).

### Selection of covariates to for analyses

#### Linear regression

We first performed a linear regression with alcohol dependence as a dependent variable to identify possible covariates (e.g. sex, age, PMI). The mean age of AD subjects was 55.65 years and was not significantly different from the age of control subjects (54.96) (Table 1). There was no significant difference in distribution of RIN and brain pH between cases and controls (Table 1). Postmortem interval (PMI) was significantly lower for the alcohol-dependent subjects.

**Table 1:** Demographic profile of alcohol-dependent and control subjects

Trait	Alcohol Dependent (*N* = 65)	Control (*N* = 73)
Male (%)	51 (78%)	60 (82%)
Mean Age (SD) (yrs)	55.65 (11.81)	54.96 (12.11)
Mean PMI (SD) (hrs)	33.66 (15.59)*	26.63 (13.25)
Brain pH (SD)	6.54 (0.23)	6.58 (0.29)
RIN (SD)	6.84 (0.96)	7.0 (1.01)

^*^P value = 0.0049

#### Variance partition analysis

**Fig. 1:**
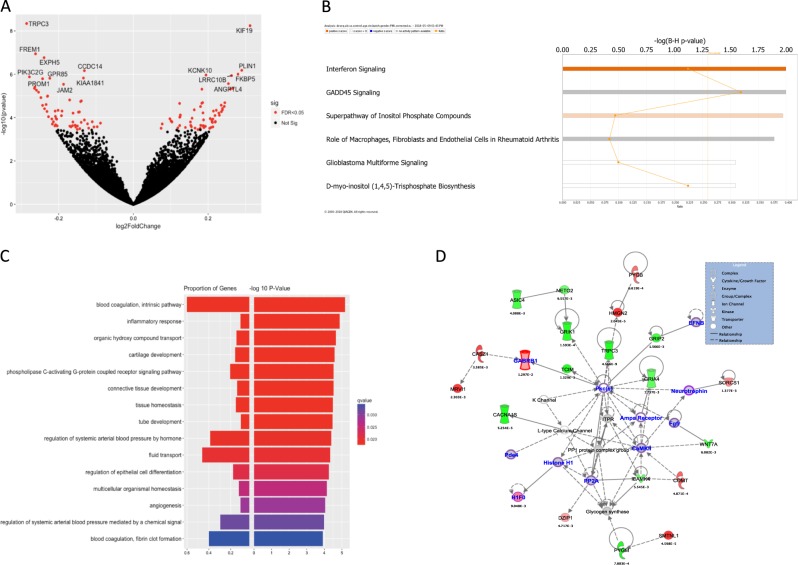
Top genes, pathways and networks from differential gene expression in DLFPC region from 68 alcoholics and 70 controls. **a** Volcano plot showing top differentially expressed genes among cases and controls. **b** The genes passing FDR threshold of 20% were inputted to IPA for pathway enrichment analysis. The figure shows some of the top pathways identified by IPA. *P* values here are from right tail Fisher’s exact test. **c** Enrichment analysis of gene ontology “biological process” terms. Color depicts the qvalues with red being the strongest evidence of enrichment. **d** Network analysis on top genes (FDR < =20%) mapped to networks involved in the neurodegenerative disorders and organismal injuries. *P* value under the gene is the uncorrected *p* value for differential expression among alcoholics and controls. The nominally significant genes in the UKBB-alc and PGC-SUD GWAS are highlighted with purple border and blue annotation

We used the variancePartition package^18^ in R to calculate the proportion of variance in RNA expression explained by known covariates such as age, gender, RIN and PMI, using the variancePartition package in R. The variancePartition^18^ package uses linear mixed model based statistical methods to quantify the contribution of multiple sources of variation and identify the covariates that required correction in the final analysis. Supplementary figure 1A shows violin plots depicting drivers of variation in gene expression data without accounting for covariates. The figure shows that sequencing batch is a major driver of variation in a large proportion of genes, while RIN and sex have large effects on only a few genes. We used the voom function in the Limma package (https://www.bioconductor.org/packages/devel/bioc/vignettes/limma/) to account for the effect of sequencing batch, RIN, age, sex and PMI on gene expression. After removing the effects of these covariates, alcohol-related phenotypes explained the largest proportion of the remaining variation in gene expression (Supplementry Figure 1B).

### Differential gene expression analysis

Gene-level analyses started with the featureCounts-derived sample-by-gene read count matrix. The basic normalization and adjustment pipeline for the expression data matrix consisted of: (i) removal of low expression genes (<1 CPM in > 50% of the individuals); (ii) differential gene expression analysis based upon adjustment for the chosen covariates. We filtered out all genes with lower expression in a substantial fraction of the cohort, with 18,463 genes with at least 1 CPM in at least 50% of the individuals; note that only these genes were carried forward in all subsequent analyses. The following design was used for the final differential expression analysis using the DeSeq2^19^ package as implimented in R: *gene expression ~ DSM4 alcohol classification* *+* *sex* *+* *age* *+* *PMI* *+* *RIN* *+* *batch*.

### Pathway analyses of differential expression

Ingenuity® Pathway Analysis (IPA®) was used to perform pathway, canonical pathways, and causal network analysis. All genes that passed the threshold of significance at 25% FDR were included in the analysis Table 2.

**Table 2:** Results of GWAS enrichment analysis in modules correlated with alcohol dependence and alcohol consumption

	RNA-Seq data (*N* = 138)						GWAS data		
	Module trait correlation						GWAS P 0.05, eQTL *P* < 5 × 10^−8^		
ID	AD	P	Audit	P	AC	P	PGC-AD	UKBB-AC	TAG-CPD
Thistle2	−0.28	9.00E-04	−0.25	3.00E-03	−0.22	9.00E-03	1.50E-02^a^	1.30E-02^a^	5.52E-01^a^
Brown4	0.18	4.00E-02	0.14	1.00E-01	0.12	1.00E-01	4.20E-03^b^	2.28E-01^b^	4.81E-03^b^

*AD* alcohol dependence, *Audit* audit scores, *AC* alcohol consumption

^a^ Permuted *P* value for the left-tail Fisher’s exact test (under-enriched)

^b^ Permuted *P* value to test right-tail Fisher’s exact test (over-enriched)

### Gene ontology analysis

Gene ontology analyses were performed using the clusterProfiler package^20^ as implemented in R. All differentially expressed genes that passed the threshold of significance at 25% FDR were included in the analysis. Results for the enrichment analysis were extracted and plotted using the ggplot2 package in R.

### Gene co-expression analysis

Scale-free co-expression networks were constructed using the weighted gene coexpression network analysis (WGCNA) package in R^15^. WGCNA provides a global perspective, emphasizing the correlation between genes to classify different molecular groupings, rather than focusing on individual genes. WGCNA defines modules using a dynamic tree-cutting algorithm based on hierarchical clustering of expression values (minimum module size = 100, cutting height = 0.99, deepSplit = TRUE). The networks were constructed at a soft power of 14 at which the scale-free topology fit index reached 0.90 (Supplementary Figure 2B). We further merged modules that had similar co-expression patterns by calculating the eigengenes and merging those having a correlation > 75% (Supplementary Figure 2C). Correlation of module eigengenes with alcohol dependence, alcohol consumption, AUDIT scores and number of years of drinking (module-trait correlation analysis) was evaluated using Spearman’s rank correlation analysis. We used the DSM4 criteria for alcohol dependence classification as provided by the New South Wales Tissue Resource Centre at the University of Sydney. For each individual in the RNA-Seq dataset a module eigen value was calculated for each module. This module eigen value was used to perform the correlation analysis of the traits (e.g. alcohol dependence, alcohol consumption and Audit scores) with each whole module. Digital deconvolution showed no significant differences in the percentage of neurons, astrocytes and microglia in the PFC of alcoholics and controls (Supplementary Figure 3)^21^; therefore we did not perform any correction for cell-type heterogeneity. Assigned modules were functionally annotated against known molecular/functional categories and pathways using Ingenuity Pathway Analysis (IPA).

### GWAS enrichment analysis

The summary statistics from a GWAS of alcohol dependence (PGC-AD) were provided by the Psychiatric Genetics Consortium Substance Use Dependence working group^22^ (Walters et al.). Summary statistics for the UKBB alcohol consumption (UKBB-AC) GWAS^23^ were provided by Dr. Toni Clarke. We also downloaded the summary statistics for Tobacco and Genetics (TAG) Consortium’s GWAS^24^ of cigarettes per day from the PGC website (https://www.med.unc.edu/pgc/results-and-downloads). SNPs from the PGC-AD and UKBB-AC studies were mapped to PFC expression quantitative trait loci (eQTLs) in 461 post-mortem brains from the Religious Orders Study and Memory and Aging Project (ROS/MAP)^25^ (Bennett et al). Enrichment analysis was performed for SNPs meeting the criteria of eQTL *P* < 5 × 10^−8^ in the ROSMAP dataset and tested for overrepresentation in GWAS of AD (PGC-AD), alcohol consumption (UKBB-AC) and TAG-CPD. Since there are a few loci that passed the genome-wide significance threshold in alcohol and smoking GWAS analysis, we tested the polygenicity of alcoholism and smoking by exploring the overenrichment in variants that passed nominal threshold of significance in these datasets. The enrichment analysis was focused on eQTLs for the genes within modules that were correlated with AD in the module-trait correlation analysis. The two modules (thistle and brown4) that showed significant enrichment (*p* ≤ 0.05) in the Fisher exact test were subjected to 100,000 permutations to report the final P value of enrichment. We also performed the gene-based analysis by Multi-marker Analysis of GenoMic Annotation (MAGMA)^26^ on summary statistics of PGC-AD, UKBB-AC and TAG-CPD GWAS using Functional Mapping and Annotation of GWAS (FUMA-GWAS)^27^. The summary statistics of this gene-based analysis were overlaid on the IPA networks to identify the genes in these networks that also have nominal to moderate evidence of genetic contributions.

## Results

### Differential expression analysis

Analysis of PFC tissue derived from 65 alcoholics and 73 controls identified 827 differentially expressed genes at 25% FDR, 298 genes at 10% FDR and 129 genes at 5% FDR (Fig. 1a, Supplemental Table 1; protein coding genes only). Transient Receptor Potential Cation Channel Subfamily C Member 3 (*TRPC3*) was the top differentially expressed gene with significantly lower expression in alcohol-dependent subjects (FC 0.82; *p* = 4.6 × 10^−9^), while Kinesin Family Member 19 (*KFM19*) showed significantly higher expression in alcohol-dependent subjects (FC 1.24; *p* = 5.7 × 10^−9^). IPA analysis of the differentially expressed genes (FDR < 25%) showed significant enrichment for pathways involved in interferon signaling, GADD45 signaling, and other immune-related pathways (Fig. 1b). Gene-ontology enrichment analysis using clusteProfiler mapped a large proportion of genes to biological processes involved in blood coagulation and fluid transport (Fig. 1c). The network analysis in IPA mapped the significant genes to networks involved in neurodegenerative disorders and organismal injury. Several genes that were part of this network were also nominally significant (*p* < 0.05) in the PGC-AD and UKBB-AC GWAS (Fig. 1d).

### Identification of gene co-expression networks and modules

**Fig. 2:**
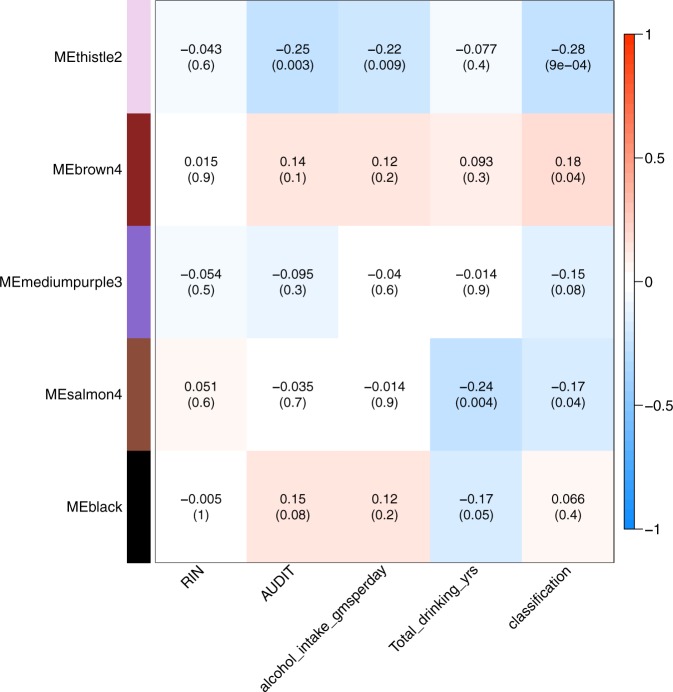
Trait module correlations with P values for the top 5 modules. WGCNA identified 27 modules, out of which 5 modules showed nominal- moderate statistical significance with any of 4 alcohol-related trait (AUDIT, alcohol consumption (gms/day), duration of drinking (years), DSM4 AD (classification). Thistle2 module also passed the multiple test correction (27 modules, 4 traits; 0.05/31 = 1.6 × 10^−3^)

After correcting for the effects of batch, age, and RIN, the hierarchical clustering of expression data from nearly 18,000 genes generated 27 different modules (Supplementary Figure 1). Trait-module correlation analyses identified five modules that were significantly correlated to at least one alcohol-related trait (Fig. 2). Of these five modules, the thistle2 module (containing 72 genes), was negatively correlated with alcohol dependence and other alcohol-related traits. The brown4 module (containing of 795 genes) was positively correlated with AD, AUDIT, alcohol consumption and duration of alcohol use.

#### Thistle2 module

**Fig. 3:**
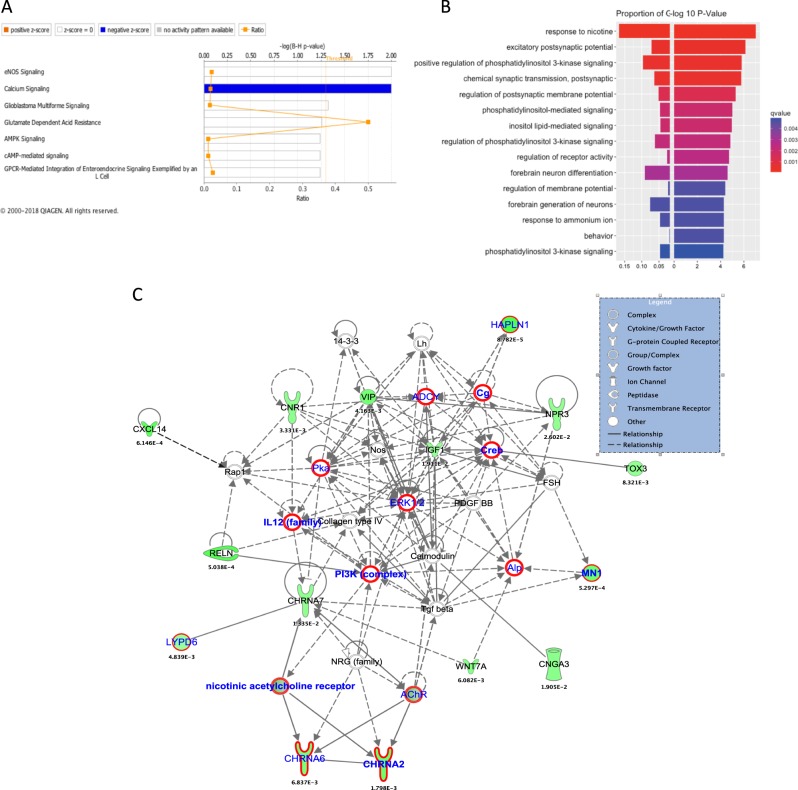
Enrichment analysis of genes in thistle2 module that are differentially expressed in alcoholics and controls. **a** More than 50% of genes in calcium signaling pathways were found to be downregulated in the thistle2 module. **b** Enrichment analysis for GO:BP terms showed downregulation of genes related to response to nicotine and postsynaptic potential. **c** Nearly 15 genes mapped to network related to amino-acid metabolism with many genes that were involved in G-protein coupled receptor signaling, calcium signaling and opioid signaling pathway. The nominally significant genes in the UKBB-alc and PGC-SUD GWAS are marked with red boundaries (*ADCY5 P* = 7.07 × 10^−7^ in UKBB-AC, *ADCY7*, *P* = 2.2 × 10^−4^ in UKBB-AC), *IL12B*, *P* = 1.1 × 10^−2^ in PGC-AD, *PIK3C2G*, *P* = 6.8 × 10^−3^ in UKBB-AC, *PIK3R4*, *P* = 3.4 × 10^−2^ in PGC-AD, *CHRNA6* in UKBB-AC *P* = 7.60 × 10^−3^, *CHRNA2* in PGC-AD *P* = 1.4 × 10^−2^, *MN1* in PGC-AD *P* = 9.1 × 10^−3^ and *HAPLN1* in UKBB-AC *P* = 1.9 × 10^−2^)

Pathway enrichment analysis of the thistle2 module showed a significant downregulation of pathways related to calcium signaling (Fig. 3a). Gene-ontology enrichment analysis using the clusterpProfiler showed significant enrichment for biological processes involved in “response to nicotine” and “excitatory postsynaptic potential” (Fig. 3b). Several genes in the thistle2 module that were significantly downregulated in the PFC of alcohol-dependent subjects. Differentially expressed genes in the thistle2 module mapped to networks involved in G-protein coupled receptor signaling, calcium signaling, and opioid signaling (Fig. 3c). Cholinergic Receptor Nicotinic Alpha subunits 6 and 2 (*CHRNA6* UKBB-AC *P* = 7.60 × 10^−3^; *CHRNA2* PGC-AD *P* = 1.4 × 10^−2^), Meningioma 1 (*MN1*, PGC-AD *P* = 9.1 × 10^−3^) and Hyaluronan And Proteoglycan Link Protein 1 (*HAPLN1*, UKBB-AC *P* = 1.9 × 10^−2^) are some exmples where differntialy expressed genes in thistle 2 module also showed some evidence of genetic contribution towards alcohol consumption or dependence.

#### Brown4 module

**Fig. 4:**
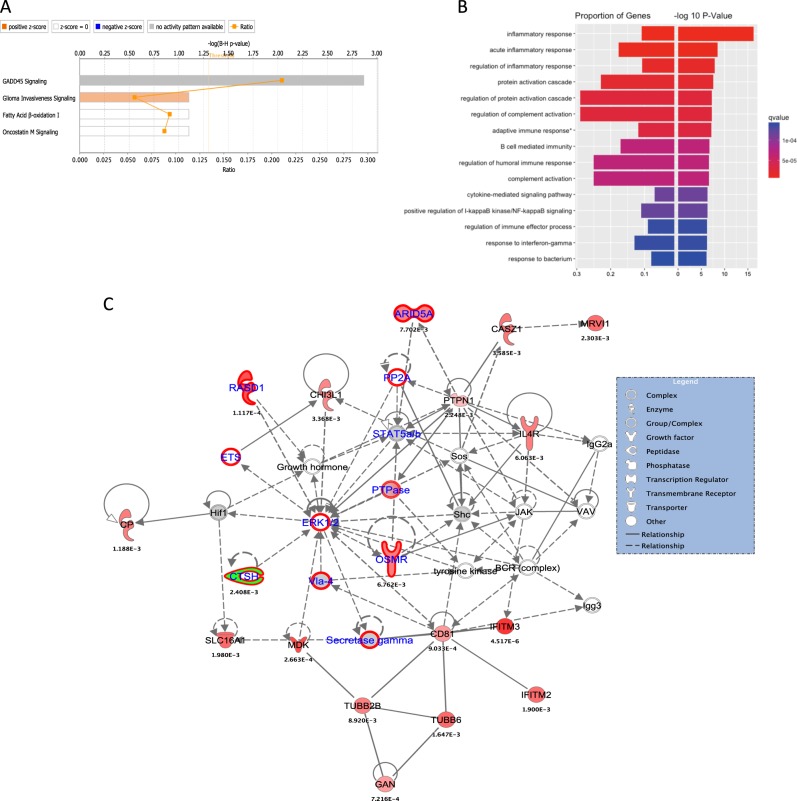
Enrichment analysis of brown4 module genes that were differentially expressed (FDR* < 0.05) among alcoholics and controls. **a** Pathway analysis showed significant upregulation of genes related immune signaling and metabolism. **b** Enrichment analysis for GO:BP terms showed enrichment of genes related to inflamatory response. **c** The genes in the brown4 module mapped to network involved in infectious and respiratory diseases. The genes that were nominally significant in the UKBB-Alc and PGC-SUD GWAS are highlighted with red boundaries

Pathway analysis for differentially expressed genes in the brown4 module showed significant enrichment for Growth Arrest and DNA Damage (GADD45) signaling and for biological processes related to the inflammatory response (Fig. 4). Other genes that were also significantly upregulated in the PFC of alcoholics mapped to networks involved in infectious and respiratory diseases.

### GWAS enrichment analysis

GWAS enrichment analysis of significant eQTLs (*P* ≤ 5 × 10^−8^) for all genes in the top 5 modules (ranked by P value in module-trait correlation analysis), showed evidence of enrichment for SNPs associated with AD (GWAS *p* ≤ 0.05) in PGC-AD and alcohol consumption in UKBB-AC datasets (Table 2). The brown4 module was also enriched for GWAS association in the TAG-CPD dataset. The thistle2 module did not show enrichment of GWAS association. Surprisingly, genes in the thistle2 modules were significantly depleted for GWAS signals in the PGC-AD and UKBB-AC GWAS analyses. This finding was confirmed by permutation analysis.

## Discussion

To our knowledge, this is the largest transcriptome analysis comparing PFC of alcohol-dependent cases and controls. The present study identified 129 genes (FDR < 0.05) that were differentially expressed in alcohol-dependent subjects (Supplementary table 1). *FKBP5*, a well studied gene that is asoociated with alcohol use^28–31^, showed increased expression in the PFC of alcohol-dependent subjects in our differential gene expression analysis (l_2_FC 0.27; *P* = 4.57 × 10^−7^). Other studies have also shown that *FKBP5* plays a role in alcohol drinking behaviors in rodents^28,29^ and humans^32^. *FKBP5* encodes FK506-binding protein 5, a glucocorticoid receptor (GR)-binding protein implicated in various psychiatric disorders and alcohol withdrawal severity^30^. Qiu and colleagues^30^ reported that *Fkbp5* KO mice exhibited increased alcohol consumption compared with wild-type mice. Another study has shown that the absence of *Fkbp5* enhances sensitivity to alcohol withdrawal in mice^33^. Recent findings also suggested that *Fkbp5* expression in mesocorticolimbic dopaminergic regions is associated with early life-stress mediated sensitivity to alcohol drinking and that there is a gene–environment interaction among *FKBP5* genotype and parent–child relationship that influences alcohol drinking.

Genes showing significant differences in expression between alcohol-dependent subjects and controls were enriched in pathways related to interferon and GADD45 signaling (Fig. 1b). Interferons are cytokines that have antiviral, antiproliferative, and immunomodulatory effects and the interferon pathway plays a critical role in human innate and adaptive immune responses^34^. Our pathway analysis results are consistent with earlier findings showing induction of innate immune genes by stress and drug abuse^35^. Furthermore, mRNA expression studies in human brain showed significant changes in expression of genes related to immune or inflammatory responses in hippocampus^7^ and nucleus accumbens^8^. The neuroinflammation associated with chronic alcohol exposure and withdrawal may be attributed to microglial activation and is associated with the neuropathology of chronic alcohol exposure^36^. Differentially expressed genes (FDR < 25%) also mapped to networks associated with neurodegenerative disorders and organismal injury (Fig. 1d). Many differentially expressed genes in this network are involved in nervous system development and function. Specifically *TRPC3* and calcium dependent protein kinase 4 (*CAMK4*) are involved in excitatory post-synaptic current while Ampa receptor, Glutamate Ionotropic receptor AMPA type subunit 4 (*GRIA4*), Calcium dependent protein kinase ii (*CaMKII*) and *CAMK4* are involved in synaptic transmission.

Although we identified several genes that were differentially expressed in the PFC of alcohol-dependent subjects, the variance explained by individual genes was very small (0.15–1%). The differential expression observed here is smaller than that reported in earlier differential expression studies of alcoholism, but it is consistent with differential expression studies of larger sample size^37^. For example, the CommonMind consortium reported similar fold changes in the differential expression study of schizophrenia and they showed that their observation is consistent with plausible models for average differential gene expression and the polygenic inheritance of schizophrenia. The polygenicity of AD has also been observed by the GWAS of alcoholism and other complex behavioral/psychiatric disorders^22,38–41^, and it was demonstrated that effect size for each individual genetic variant is very small. Studies that used a co-expression network approach also showed that alcohol dependence is shaped, in part, by persistent alterations in networks of co-expressed genes that collectively mediate excessive drinking and other alcohol-dependent phenotypes^8,9^. These and other studies also demonstrated that the gene network structure is significantly correlated with lifetime alcohol consumption in addition to an overall loss in network structure; furthermore, the neurobiology of alcohol dependence may be due to altered covariation of gene modules, rather than discrete changes in differentially expressed genes across the transcriptome^9,13^.

Trait-module correlation analysis for the thistle2 module showed a significant negative correlation with alcohol dependence (−0.28, *P* = 9.0 × 10^−4^), alcohol consumption (−0.22, *P* = 9.0 × 10^−3^), and AUDIT score (−0.25, *P* = 3.0 × 10^−3^), while the brown4 module showed a positive correlation (0.18, *P* = 4.0 × 10^−2^) with alcohol dependence (Fig. 2; Table 2). The salmon4 module was associated with the total number of drinking years (−0.24, *P* = 4.0 × 10^−3^), independent of the age of the subjects. Genes in the thistle2 module were significantly downregulated in the PFC from alcoholics. Many genes in the thistle2 module mapped to networks involved in opioid signaling and nicotine response, highlighting the importance of this module in addiction-related traits. Pathway analysis showed that all genes that overlapped with genes involved in calcium signaling were significantly downregulated (Fig. 3a). Acute ethanol exposure has been shown to inhibit Ca^2+^ currents induced by PKC-dependent phosphorylation of mGluR5 in neurons^42^. Early studies in PC12 cell cultures also showed that ethanol has a significant inhibitory effect on the influx of Ca^2+^ through L-type voltage-gated Ca^2+^ channels^43^. Alcohol exposure also modulates Ca^2+^ signaling between astrocytes and neurons^44^ (Warden et al.), and Ca^2+^ acts as a second messenger that controls multiple processes in immune cells, including chemotaxis and secretion of pro- and anti-inflammatory cytokines. Our analyses provide further evidence that alcohol exposure alters Ca^2+^ signaling in the brains of alcoholics and could potentially alter communication between neurons and brain immune cells. Another module that correlated with alcohol dependence, brown4, was also enriched in immune response and infectious diseases, providing additional evidence for the role of the neuroimmune system in the etiology of alcohol dependence. Some of the differentially expressed genes in this network were also statistical significant in the gene-based tests (*RASD1*, UKBB-AC, *P* = 1.64 × 10^−5^ and *ARID5A*, UKBB-AC, P 1.4 × 10^−3^). The differentially expressed *FKBP5* gene was also part of the brown module, but it was not identified as hub gene according to intra-modular connectivity (supplementary table 2).

Enrichment analysis of nominally significant GWAS variants (*p* < 0.05) that were also eQTLs (*p* < 5 × 10^−8^) for genes in the thistle2 module showed significant under enrichment in the two-tail Fisher test (Table 2). The under-enrichment remained significant even after 100,000 permutations. This might be due to the small size of this module (*N* = 72 genes). Although some of the differntialy expressed genes were significant in the gene-based tests performed in UKBB-AC and PGC-AD datasets using MAGMA (*CHRNA6*, *CHRNA2*, *MN1* and *HAPLN1*). In the calcium signaling network (Fig. 3c), a few genes that were not part of the thistle2 module, but were essential to create network connections, were also found to be significant (3.4 × 10^−2^ ≤ *P* ≥ 4.8 × 10^−2^) in the gene-based tests (circled in red; Fig. 4c). This suggests possible gene–environment (alcohol exposure) interactions in the etiology of alcohol dependence. This also reinforces the need for multi-omics data to understand a complex disorder like alcoholism. eQTLs for genes in the brown4 module (*N* = 726 genes) were significantly enriched for GWAS signals (*P* = 4.2 × 10^−3^) in the PGC-AD GWAS. Interestingly this module was also positively correlated with alcohol dependence (0.18, *P* = 4.0 × 10^−2^) in trait-module correlation analysis.

Because of limited availability of human post-mortem tissue with DSM4 alcohol dependence phenotype, we tried to look for validation in rodent RNA-expression datasets (Supplementary methods; supplementary table 2). The hub genes identified in present analysis were found to be significant enriched for association signals in the rodents. This observation adds to the validity of hub genes in the identified modules.

In the present study, we focused on integrating the genomic information to transcriptomic data to identify gene (genetic background) × environment (alcohol exposure) interactions in the etiology of alcohol use disorders. As mentioned in the discussion we identified that genes that have altered expression due to alcohol exposure interact with risk genes (GWAS) to increase an individual’s risk of becoming dependent on alcohol. So, to translate these findings in animals, one has to mimic expression of hub genes as well as the risk gene to alter the pathways associated with alcoholism. We are also reporting the direction of effect of the differential expression. That should provide information that can be used to see whether knock-down or overexpression of key genes alters risk for AUD phenotypes in models. Also, the replication of the modules in rodent models indicates which models might be useful to study the effects of dysregulation in these models.

Multiple lines of evidence derived from this study allowed us to prioritize the genes altered by exposure to alcohol. The gene co-expression network analysis also identified networks of genes altered in alcohol-dependent subjects. Further support for our findings comes from work showing that many genes in these networks were also associated with alcohol dependence and alcohol consumption in large GWAS study cohorts. This systematic exploration of transcriptomic organization in the PFC from alcoholics provides further support for the role of the neuroimmune system in alcohol dependence. The biological pathways and networks of genes identified in the current study will help prioritize genes for functional studies and may help advance targeted treatment approaches for alcohol use disorders.

## Supplementary information

Supplementary methodology and figure headings

Supplementary Table 1

Supplementary Table 2

Supplementary Figure 1

Supplementary Figure 2

Supplementary Figure 3

Supplementary Figure 4

## Data Availability

Data sharing policy: RNA-Seq data for alcohol dependence cases and controls will be available through GEO. All the summary statistics for differential expression will also be posted at INIA and COGA’s homepage (and Shiny web app) and will be freely available to download after publication is online. Shiny webapp link for entire summary statistics: https://lcad.shinyapps.io/coga-inia/ (Access code: coga-inia).
