# Supplementary material for: Analysis of whole genome-transcriptomic organization in brain to identify genes associated with alcoholism
Source: Transl Psychiatry. 2019 Feb 14;9:89. doi: 10.1038/s41398-019-0384-y (PMC6376002; doi:10.1038/s41398-019-0384-y)
Supplement: Supplementary file 2 — Supplementary Table 1 [file 41398_2019_384_MOESM2_ESM.pdf]

| Supp table 1: Differntialy expressed genes in brain of AD subjects at 25% FDR | | | | | | | |
|---|---|---|---|---|---|---|---|
| | | | | | | | |
| **ID** | **Gene** | **log2FoldCha** | **FC** | **lfcSE** | **pvalue** | **padj** | **Module Color** |
| ENSG00000138741 | TRPC3 | -0.29 | 0.82 | 0.05 | 4.57E-09 | 4.16E-05 | brown |
| ENSG00000196169 | KIF19 | 0.31 | 1.24 | 0.05 | 5.74E-09 | 4.16E-05 | blue |
| ENSG00000164946 | FREM1 | -0.26 | 0.83 | 0.05 | 1.14E-07 | 5.49E-04 | thistle2 |
| ENSG00000110723 | EXPH5 | -0.24 | 0.85 | 0.05 | 1.72E-07 | 6.22E-04 | thistle2 |
| ENSG00000166819 | PLIN1 | 0.29 | 1.22 | 0.06 | 6.58E-07 | 1.65E-03 | black |
| ENSG00000175455 | CCDC14 | -0.13 | 0.91 | 0.03 | 6.84E-07 | 1.65E-03 | paleturquoise |
| ENSG00000096060 | FKBP5 | 0.28 | 1.21 | 0.06 | 9.96E-07 | 1.80E-03 | brown4 |
| ENSG00000100433 | KCNK10 | 0.19 | 1.14 | 0.04 | 1.08E-06 | 1.80E-03 | blue |
| ENSG00000204950 | LRRC10B | 0.26 | 1.20 | 0.05 | 1.16E-06 | 1.80E-03 | grey60 |
| ENSG00000139144 | PIK3C2G | -0.28 | 0.82 | 0.06 | 1.32E-06 | 1.80E-03 | grey |
| ENSG00000162929 | KIAA1841 | -0.13 | 0.91 | 0.03 | 1.50E-06 | 1.80E-03 | brown |
| ENSG00000164604 | GPR85 | -0.22 | 0.86 | 0.05 | 1.54E-06 | 1.80E-03 | brown |
| ENSG00000007062 | PROM1 | -0.24 | 0.84 | 0.05 | 1.61E-06 | 1.80E-03 | brown |
| ENSG00000167772 | ANGPTL4 | 0.25 | 1.19 | 0.05 | 2.72E-06 | 2.81E-03 | brown4 |
| ENSG00000154721 | JAM2 | -0.19 | 0.88 | 0.04 | 2.91E-06 | 2.81E-03 | black |
| ENSG00000162654 | GBP4 | -0.26 | 0.83 | 0.06 | 3.94E-06 | 3.56E-03 | brown |
| ENSG00000111404 | RERGL | -0.26 | 0.83 | 0.06 | 4.50E-06 | 3.56E-03 | brown |
| ENSG00000142089 | IFITM3 | 0.27 | 1.20 | 0.06 | 4.52E-06 | 3.56E-03 | brown4 |
| ENSG00000106714 | CNTNAP3 | 0.18 | 1.14 | 0.04 | 4.90E-06 | 3.56E-03 | blue |
| ENSG00000183856 | IQGAP3 | 0.26 | 1.20 | 0.06 | 4.91E-06 | 3.56E-03 | grey |
| ENSG00000142449 | FBN3 | -0.26 | 0.84 | 0.06 | 5.47E-06 | 3.77E-03 | grey60 |
| ENSG00000153822 | KCNJ16 | -0.25 | 0.84 | 0.06 | 6.77E-06 | 4.45E-03 | black |
| ENSG00000170959 | DCDC1 | -0.23 | 0.85 | 0.05 | 1.01E-05 | 6.33E-03 | turquoise |
| ENSG00000117009 | KMO | -0.23 | 0.85 | 0.05 | 1.05E-05 | 6.33E-03 | mediumpurple3 |
| ENSG00000107562 | CXCL12 | -0.25 | 0.84 | 0.06 | 1.20E-05 | 6.95E-03 | brown |
| ENSG00000206432 | TMEM200C | -0.17 | 0.89 | 0.04 | 1.53E-05 | 8.54E-03 | lightsteelblue1 |
| ENSG00000138336 | TET1 | -0.14 | 0.91 | 0.03 | 1.70E-05 | 9.12E-03 | paleturquoise |
| ENSG00000172878 | METAP1D | -0.14 | 0.91 | 0.03 | 1.82E-05 | 9.43E-03 | turquoise |
| ENSG00000198830 | HMGN2 | 0.19 | 1.14 | 0.04 | 2.04E-05 | 9.98E-03 | paleturquoise |
| ENSG00000102359 | SRPX2 | 0.24 | 1.18 | 0.06 | 2.07E-05 | 9.98E-03 | brown4 |
| ENSG00000129353 | SLC44A2 | 0.18 | 1.14 | 0.04 | 2.19E-05 | 1.01E-02 | brown4 |
| ENSG00000198408 | MGEA5 | 0.12 | 1.09 | 0.03 | 2.23E-05 | 1.01E-02 | darkgrey |
| ENSG00000205221 | VIT | -0.23 | 0.85 | 0.06 | 2.50E-05 | 1.10E-02 | brown |
| ENSG00000141736 | ERBB2 | 0.23 | 1.18 | 0.06 | 2.70E-05 | 1.14E-02 | brown4 |
| ENSG00000183090 | FREM3 | -0.24 | 0.84 | 0.06 | 2.75E-05 | 1.14E-02 | mediumpurple3 |
| ENSG00000132470 | ITGB4 | 0.24 | 1.18 | 0.06 | 3.06E-05 | 1.21E-02 | brown4 |
| ENSG00000141314 | RHBDL3 | 0.18 | 1.13 | 0.04 | 3.10E-05 | 1.21E-02 | lightsteelblue1 |
| ENSG00000147872 | PLIN2 | 0.24 | 1.18 | 0.06 | 3.82E-05 | 1.46E-02 | brown4 |
| ENSG00000143341 | HMCN1 | -0.24 | 0.85 | 0.06 | 3.93E-05 | 1.46E-02 | lightgreen |
| ENSG00000111696 | NT5DC3 | 0.16 | 1.12 | 0.04 | 4.40E-05 | 1.55E-02 | lightsteelblue1 |
| ENSG00000124145 | SDC4 | 0.24 | 1.18 | 0.06 | 4.45E-05 | 1.55E-02 | black |
| ENSG00000214872 | SMTNL1 | 0.22 | 1.17 | 0.05 | 4.60E-05 | 1.55E-02 | turquoise |
| ENSG00000165899 | OTOGL | -0.20 | 0.87 | 0.05 | 4.61E-05 | 1.55E-02 | lightsteelblue1 |
| ENSG00000147647 | DPYS | -0.19 | 0.88 | 0.05 | 5.03E-05 | 1.59E-02 | lightsteelblue1 |
| ENSG00000074410 | CA12 | 0.21 | 1.16 | 0.05 | 5.04E-05 | 1.59E-02 | brown4 |
| ENSG00000116711 | PLA2G4A | -0.21 | 0.86 | 0.05 | 5.05E-05 | 1.59E-02 | brown |
| ENSG00000081248 | CACNA1S | -0.23 | 0.85 | 0.06 | 5.25E-05 | 1.61E-02 | grey60 |

| | | | | | | | |
|---|---|---|---|---|---|---|---|
| ENSG00000116717 | GADD45A | 0.24 | 1.18 | 0.06 | 5.34E-05 | 1.61E-02 | brown4 |
| ENSG00000120756 | PLS1 | -0.14 | 0.90 | 0.04 | 5.49E-05 | 1.62E-02 | lightsteelblue1 |
| ENSG00000048052 | HDAC9 | -0.15 | 0.90 | 0.04 | 5.62E-05 | 1.63E-02 | lightsteelblue1 |
| ENSG00000137857 | DUOX1 | 0.19 | 1.14 | 0.05 | 6.08E-05 | 1.71E-02 | yellow |
| ENSG00000113369 | ARRDC3 | 0.23 | 1.17 | 0.06 | 6.18E-05 | 1.71E-02 | brown4 |
| ENSG00000244694 | PTCHD4 | -0.16 | 0.89 | 0.04 | 6.26E-05 | 1.71E-02 | mediumpurple3 |
| ENSG00000050165 | DKK3 | 0.13 | 1.09 | 0.03 | 7.62E-05 | 2.04E-02 | brown |
| ENSG00000122863 | CHST3 | 0.23 | 1.17 | 0.06 | 7.85E-05 | 2.07E-02 | brown4 |
| ENSG00000187193 | MT1X | 0.22 | 1.16 | 0.06 | 8.09E-05 | 2.09E-02 | brown4 |
| ENSG00000154736 | ADAMTS5 | -0.19 | 0.88 | 0.05 | 8.23E-05 | 2.09E-02 | navajowhite2 |
| ENSG00000145681 | HAPLN1 | -0.21 | 0.87 | 0.05 | 8.78E-05 | 2.19E-02 | thistle2 |
| ENSG00000010322 | NISCH | 0.12 | 1.09 | 0.03 | 9.15E-05 | 2.21E-02 | yellow |
| ENSG00000188729 | OSTN | -0.23 | 0.85 | 0.06 | 9.15E-05 | 2.21E-02 | brown |
| ENSG00000158480 | SPATA2 | 0.12 | 1.09 | 0.03 | 9.68E-05 | 2.30E-02 | yellow |
| ENSG00000060656 | PTPRU | 0.16 | 1.11 | 0.04 | 0.00010754 | 2.51E-02 | yellow |
| ENSG00000110092 | CCND1 | -0.21 | 0.87 | 0.05 | 0.00011148 | 2.52E-02 | lightgreen |
| ENSG00000108551 | RASD1 | 0.21 | 1.15 | 0.05 | 0.00011168 | 2.52E-02 | brown4 |
| ENSG00000173011 | TADA2B | 0.10 | 1.07 | 0.03 | 0.00011371 | 2.52E-02 | yellow |
| ENSG00000124205 | EDN3 | -0.22 | 0.86 | 0.06 | 0.0001164 | 2.52E-02 | brown |
| ENSG00000164104 | HMGB2 | 0.21 | 1.16 | 0.06 | 0.00011701 | 2.52E-02 | brown4 |
| ENSG00000139304 | PTPRQ | -0.21 | 0.87 | 0.05 | 0.00011827 | 2.52E-02 | turquoise |
| ENSG00000131187 | F12 | 0.19 | 1.14 | 0.05 | 0.00012814 | 2.68E-02 | grey60 |
| ENSG00000128578 | STRIP2 | -0.13 | 0.91 | 0.03 | 0.00012947 | 2.68E-02 | mediumpurple3 |
| ENSG00000141469 | SLC14A1 | 0.21 | 1.16 | 0.06 | 0.00013691 | 2.79E-02 | black |
| ENSG00000155287 | SLC25A28 | 0.10 | 1.07 | 0.03 | 0.00014467 | 2.90E-02 | blue |
| ENSG00000144908 | ALDH1L1 | 0.22 | 1.16 | 0.06 | 0.00015171 | 2.90E-02 | brown4 |
| ENSG00000183323 | CCDC125 | -0.11 | 0.93 | 0.03 | 0.00015593 | 2.90E-02 | blue |
| ENSG00000162367 | TAL1 | -0.21 | 0.86 | 0.06 | 0.00015631 | 2.90E-02 | lightcyan |
| ENSG00000175104 | TRAF6 | 0.10 | 1.07 | 0.03 | 0.00015828 | 2.90E-02 | darkgrey |
| ENSG00000122574 | WIPF3 | 0.15 | 1.11 | 0.04 | 0.00015889 | 2.90E-02 | yellow |
| ENSG00000171189 | GRIK1 | -0.16 | 0.89 | 0.04 | 0.00015929 | 2.90E-02 | thistle2 |
| ENSG00000102362 | SYTL4 | 0.22 | 1.16 | 0.06 | 0.00016008 | 2.90E-02 | brown4 |
| ENSG00000151929 | BAG3 | 0.21 | 1.16 | 0.06 | 0.00016021 | 2.90E-02 | brown4 |
| ENSG00000152818 | UTRN | -0.14 | 0.91 | 0.04 | 0.00016545 | 2.93E-02 | lightgreen |
| ENSG00000111907 | TPD52L1 | 0.21 | 1.16 | 0.06 | 0.00016585 | 2.93E-02 | black |
| ENSG00000138246 | DNAJC13 | -0.09 | 0.94 | 0.02 | 0.0001791 | 3.12E-02 | darkgrey |
| ENSG00000123473 | STIL | -0.12 | 0.92 | 0.03 | 0.00019321 | 3.33E-02 | brown |
| ENSG00000101413 | RPRD1B | 0.08 | 1.05 | 0.02 | 0.00019661 | 3.34E-02 | brown4 |
| ENSG00000141682 | PMAIP1 | -0.22 | 0.86 | 0.06 | 0.00019847 | 3.34E-02 | navajowhite2 |
| ENSG00000118777 | ABCG2 | -0.21 | 0.87 | 0.06 | 0.00021336 | 3.55E-02 | brown |
| ENSG00000120907 | ADRA1A | -0.16 | 0.90 | 0.04 | 0.00022169 | 3.63E-02 | salmon4 |
| ENSG00000178015 | GPR150 | 0.18 | 1.13 | 0.05 | 0.00022565 | 3.63E-02 | brown |
| ENSG00000058866 | DGKG | 0.15 | 1.11 | 0.04 | 0.00022725 | 3.63E-02 | brown4 |
| ENSG00000174576 | NPAS4 | 0.12 | 1.09 | 0.03 | 0.00023018 | 3.63E-02 | darkslateblue |
| ENSG00000225828 | FAM229A | 0.19 | 1.14 | 0.05 | 0.00023524 | 3.63E-02 | yellow |
| ENSG00000112706 | IMPG1 | -0.16 | 0.89 | 0.04 | 0.00023571 | 3.63E-02 | darkgrey |
| ENSG00000103742 | IGDCC4 | 0.19 | 1.14 | 0.05 | 0.00023789 | 3.63E-02 | brown4 |
| ENSG00000136297 | MMD2 | -0.21 | 0.86 | 0.06 | 0.00024055 | 3.63E-02 | salmon4 |
| ENSG00000166035 | LIPC | -0.20 | 0.87 | 0.06 | 0.00024097 | 3.63E-02 | grey |
| ENSG00000173085 | COQ2 | -0.15 | 0.90 | 0.04 | 0.00024339 | 3.63E-02 | paleturquoise |

| | | | | | | | |
|---|---|---|---|---|---|---|---|
| ENSG00000186160 | CYP4Z1 | -0.17 | 0.89 | 0.05 | 0.0002476 | 3.66E-02 | turquoise |
| ENSG00000163346 | PBXIP1 | 0.19 | 1.14 | 0.05 | 0.00025246 | 3.69E-02 | black |
| ENSG00000126003 | PLAGL2 | 0.16 | 1.11 | 0.04 | 0.00026342 | 3.72E-02 | yellow |
| ENSG00000135537 | LACE1 | -0.10 | 0.94 | 0.03 | 0.00026385 | 3.72E-02 | darkgrey |
| ENSG00000110492 | MDK | 0.21 | 1.16 | 0.06 | 0.00026626 | 3.72E-02 | brown4 |
| ENSG00000176273 | SLC35G1 | -0.17 | 0.89 | 0.05 | 0.00026642 | 3.72E-02 | brown |
| ENSG00000071575 | TRIB2 | -0.15 | 0.90 | 0.04 | 0.00026958 | 3.72E-02 | darkslateblue |
| ENSG00000132334 | PTPRE | -0.11 | 0.93 | 0.03 | 0.00026971 | 3.72E-02 | brown |
| ENSG00000130821 | SLC6A8 | 0.14 | 1.10 | 0.04 | 0.00028428 | 3.87E-02 | blue |
| ENSG00000160862 | AZGP1 | 0.21 | 1.15 | 0.06 | 0.0002859 | 3.87E-02 | blue |
| ENSG00000115112 | TFCP2L1 | 0.19 | 1.14 | 0.05 | 0.00028938 | 3.88E-02 | brown4 |
| ENSG00000156030 | ELMSAN1 | 0.12 | 1.09 | 0.03 | 0.00029571 | 3.93E-02 | brown4 |
| ENSG00000120885 | CLU | 0.16 | 1.12 | 0.05 | 0.00030398 | 3.98E-02 | brown4 |
| ENSG00000151470 | C4orf33 | -0.12 | 0.92 | 0.03 | 0.00030588 | 3.98E-02 | brown |
| ENSG00000087274 | ADD1 | 0.07 | 1.05 | 0.02 | 0.00030907 | 3.98E-02 | blue |
| ENSG00000198574 | SH2D1B | 0.20 | 1.15 | 0.06 | 0.00031042 | 3.98E-02 | darkorange2 |
| ENSG00000018280 | SLC11A1 | 0.18 | 1.13 | 0.05 | 0.00031467 | 4.00E-02 | brown4 |
| ENSG00000101134 | DOK5 | -0.14 | 0.91 | 0.04 | 0.00032363 | 4.07E-02 | lightsteelblue1 |
| ENSG00000170396 | ZNF804A | -0.15 | 0.90 | 0.04 | 0.00035004 | 4.37E-02 | lightsteelblue1 |
| ENSG00000115758 | ODC1 | 0.17 | 1.13 | 0.05 | 0.00035701 | 4.42E-02 | brown |
| ENSG00000172399 | MYOZ2 | 0.18 | 1.13 | 0.05 | 0.00036533 | 4.48E-02 | brown |
| ENSG00000240038 | AMY2B | -0.11 | 0.93 | 0.03 | 0.00037326 | 4.51E-02 | darkgrey |
| ENSG00000120158 | RCL1 | 0.12 | 1.08 | 0.03 | 0.00037687 | 4.51E-02 | brown4 |
| ENSG00000049540 | ELN | -0.20 | 0.87 | 0.06 | 0.00037739 | 4.51E-02 | blue |
| ENSG00000143494 | VASH2 | 0.16 | 1.11 | 0.04 | 0.00038871 | 4.61E-02 | turquoise |
| ENSG00000164035 | EMCN | -0.19 | 0.87 | 0.05 | 0.00039468 | 4.63E-02 | lightcyan |
| ENSG00000203668 | CHML | -0.14 | 0.91 | 0.04 | 0.00039624 | 4.63E-02 | lightsteelblue1 |
| ENSG00000162621 | LRRC53 | -0.19 | 0.88 | 0.05 | 0.00040625 | 4.70E-02 | grey |
| ENSG00000170365 | SMAD1 | 0.12 | 1.09 | 0.03 | 0.0004182 | 4.80E-02 | brown4 |
| ENSG00000152583 | SPARCL1 | 0.15 | 1.11 | 0.04 | 0.00042422 | 4.82E-02 | brown4 |
| ENSG00000182508 | LHFPL1 | -0.20 | 0.87 | 0.06 | 0.000426 | 4.82E-02 | black |
| ENSG00000111405 | ENDOU | 0.20 | 1.15 | 0.06 | 0.00045338 | 5.09E-02 | black |
| ENSG00000099968 | BCL2L13 | 0.09 | 1.07 | 0.03 | 0.00045948 | 5.12E-02 | brown |
| ENSG00000168743 | NPNT | -0.18 | 0.88 | 0.05 | 0.00046405 | 5.12E-02 | lightgreen |
| ENSG00000144306 | SCRN3 | -0.11 | 0.93 | 0.03 | 0.00046709 | 5.12E-02 | paleturquoise |
| ENSG00000139329 | LUM | -0.20 | 0.87 | 0.06 | 0.00047643 | 5.18E-02 | lightgreen |
| ENSG00000137288 | UQCC2 | -0.11 | 0.93 | 0.03 | 0.00048321 | 5.22E-02 | grey60 |
| ENSG00000093010 | COMT | 0.14 | 1.10 | 0.04 | 0.00048709 | 5.22E-02 | blue |
| ENSG00000081059 | TCF7 | 0.17 | 1.12 | 0.05 | 0.00049297 | 5.22E-02 | yellow |
| ENSG00000132793 | LPIN3 | 0.19 | 1.14 | 0.06 | 0.000495 | 5.22E-02 | brown4 |
| ENSG00000180287 | PLD5 | -0.15 | 0.90 | 0.04 | 0.00049757 | 5.22E-02 | thistle2 |
| ENSG00000189056 | RELN | -0.20 | 0.87 | 0.06 | 0.00050383 | 5.23E-02 | thistle2 |
| ENSG00000015133 | CCDC88C | -0.18 | 0.88 | 0.05 | 0.00050626 | 5.23E-02 | grey60 |
| ENSG00000169184 | MN1 | -0.17 | 0.89 | 0.05 | 0.00052968 | 5.44E-02 | thistle2 |
| ENSG00000095015 | MAP3K1 | -0.12 | 0.92 | 0.03 | 0.0005399 | 5.50E-02 | paleturquoise |
| ENSG00000131378 | RFTN1 | -0.13 | 0.91 | 0.04 | 0.00054922 | 5.53E-02 | grey60 |
| ENSG00000079819 | EPB41L2 | -0.10 | 0.93 | 0.03 | 0.00055007 | 5.53E-02 | mediumpurple3 |
| ENSG00000113240 | CLK4 | -0.11 | 0.93 | 0.03 | 0.00055655 | 5.56E-02 | brown |
| ENSG00000008394 | MGST1 | 0.20 | 1.15 | 0.06 | 0.00057177 | 5.64E-02 | brown4 |
| ENSG00000148600 | CDHR1 | 0.19 | 1.14 | 0.06 | 0.00057271 | 5.64E-02 | yellow |

| | | | | | | | |
|---|---|---|---|---|---|---|---|
| ENSG00000156587 | UBE2L6 | 0.15 | 1.11 | 0.04 | 0.00057898 | 5.66E-02 | palevioletred3 |
| ENSG00000074803 | SLC12A1 | -0.19 | 0.88 | 0.06 | 0.00058282 | 5.66E-02 | lightsteelblue1 |
| ENSG00000016391 | CHDH | 0.15 | 1.11 | 0.04 | 0.00058839 | 5.68E-02 | black |
| ENSG00000211452 | DIO1 | 0.19 | 1.14 | 0.05 | 0.00061319 | 5.85E-02 | turquoise |
| ENSG00000145824 | CXCL14 | -0.19 | 0.88 | 0.06 | 0.00061462 | 5.85E-02 | thistle2 |
| ENSG00000183091 | NEB | -0.16 | 0.89 | 0.05 | 0.00063305 | 5.99E-02 | grey |
| ENSG00000007384 | RHBDF1 | 0.17 | 1.12 | 0.05 | 0.0006535 | 6.07E-02 | brown4 |
| ENSG00000106025 | TSPAN12 | -0.18 | 0.88 | 0.05 | 0.00065674 | 6.07E-02 | black |
| ENSG00000165685 | TMEM52B | -0.20 | 0.87 | 0.06 | 0.00066005 | 6.07E-02 | brown |
| ENSG00000100994 | PYGB | 0.13 | 1.09 | 0.04 | 0.00066191 | 6.07E-02 | darkolivegreen |
| ENSG00000180638 | SLC47A2 | 0.18 | 1.14 | 0.05 | 0.0006626 | 6.07E-02 | yellow |
| ENSG00000154330 | PGM5 | -0.19 | 0.87 | 0.06 | 0.00067169 | 6.08E-02 | lightgreen |
| ENSG00000140022 | STON2 | 0.19 | 1.14 | 0.06 | 0.0006758 | 6.08E-02 | black |
| ENSG00000174175 | SELP | 0.20 | 1.15 | 0.06 | 0.00067664 | 6.08E-02 | turquoise |
| ENSG00000004799 | PDK4 | 0.19 | 1.14 | 0.06 | 0.00068053 | 6.08E-02 | brown4 |
| ENSG00000161395 | PGAP3 | 0.11 | 1.08 | 0.03 | 0.00069704 | 6.12E-02 | darkolivegreen |
| ENSG00000101203 | COL20A1 | -0.18 | 0.88 | 0.05 | 0.00070022 | 6.12E-02 | tan |
| ENSG00000137766 | UNC13C | -0.13 | 0.91 | 0.04 | 0.00070938 | 6.12E-02 | brown |
| ENSG00000163453 | IGFBP7 | 0.18 | 1.13 | 0.05 | 0.00071416 | 6.12E-02 | brown4 |
| ENSG00000164512 | ANKRD55 | -0.17 | 0.89 | 0.05 | 0.00071687 | 6.12E-02 | thistle2 |
| ENSG00000122477 | LRRC39 | -0.12 | 0.92 | 0.04 | 0.00072138 | 6.12E-02 | brown |
| ENSG00000261609 | GAN | 0.12 | 1.08 | 0.03 | 0.00072161 | 6.12E-02 | brown4 |
| ENSG00000136436 | CALCOCO2 | 0.14 | 1.10 | 0.04 | 0.00072473 | 6.12E-02 | brown4 |
| ENSG00000111846 | GCNT2 | -0.17 | 0.89 | 0.05 | 0.0007308 | 6.12E-02 | thistle2 |
| ENSG00000172554 | SNTG2 | 0.20 | 1.15 | 0.06 | 0.00073235 | 6.12E-02 | grey |
| ENSG00000144366 | GULP1 | -0.14 | 0.91 | 0.04 | 0.00073323 | 6.12E-02 | lightsteelblue1 |
| ENSG00000197943 | PLCG2 | -0.18 | 0.88 | 0.05 | 0.00073529 | 6.12E-02 | lightcyan |
| ENSG00000140545 | MFGE8 | -0.17 | 0.89 | 0.05 | 0.00074105 | 6.12E-02 | grey60 |
| ENSG00000163808 | KIF15 | -0.14 | 0.91 | 0.04 | 0.00074403 | 6.12E-02 | darkgrey |
| ENSG00000143819 | EPHX1 | 0.18 | 1.13 | 0.05 | 0.00075017 | 6.12E-02 | brown4 |
| ENSG00000086717 | PPEF1 | -0.18 | 0.88 | 0.05 | 0.00075211 | 6.12E-02 | brown |
| ENSG00000120318 | ARAP3 | 0.19 | 1.14 | 0.06 | 0.00078495 | 6.27E-02 | tan |
| ENSG00000130222 | GADD45G | 0.19 | 1.14 | 0.06 | 0.00078691 | 6.27E-02 | brown4 |
| ENSG00000003989 | SLC7A2 | 0.20 | 1.15 | 0.06 | 0.00078692 | 6.27E-02 | brown4 |
| ENSG00000068976 | PYGM | -0.19 | 0.87 | 0.06 | 0.00078833 | 6.27E-02 | salmon4 |
| ENSG00000198342 | ZNF442 | 0.19 | 1.14 | 0.06 | 0.00080356 | 6.32E-02 | brown4 |
| ENSG00000150630 | VEGFC | -0.19 | 0.88 | 0.06 | 0.00080402 | 6.32E-02 | lightgreen |
| ENSG00000140522 | RLBP1 | -0.19 | 0.88 | 0.06 | 0.00081203 | 6.34E-02 | salmon4 |
| ENSG00000051825 | MPHOSPH9 | -0.08 | 0.95 | 0.02 | 0.0008184 | 6.34E-02 | turquoise |
| ENSG00000000003 | TSPAN6 | 0.18 | 1.13 | 0.05 | 0.00082096 | 6.34E-02 | brown4 |
| ENSG00000042286 | AIFM2 | 0.15 | 1.11 | 0.04 | 0.00082286 | 6.34E-02 | tan |
| ENSG00000143390 | RFX5 | -0.11 | 0.93 | 0.03 | 0.00083158 | 6.37E-02 | brown |
| ENSG00000237172 | B3GNT9 | 0.17 | 1.12 | 0.05 | 0.00084681 | 6.45E-02 | blue |
| ENSG00000143554 | SLC27A3 | 0.16 | 1.12 | 0.05 | 0.00085366 | 6.47E-02 | brown4 |
| ENSG00000126709 | IFI6 | 0.19 | 1.14 | 0.06 | 0.00086261 | 6.50E-02 | palevioletred3 |
| ENSG00000229894 | GK3P | 0.18 | 1.13 | 0.05 | 0.0008801 | 6.58E-02 | turquoise |
| ENSG00000196510 | ANAPC7 | -0.09 | 0.94 | 0.03 | 0.00088223 | 6.58E-02 | brown |
| ENSG00000147852 | VLDLR | -0.14 | 0.91 | 0.04 | 0.00089301 | 6.62E-02 | brown |
| ENSG00000172197 | MBOAT1 | -0.19 | 0.87 | 0.06 | 0.00089613 | 6.62E-02 | blue |
| ENSG00000110651 | CD81 | 0.12 | 1.09 | 0.04 | 0.00090329 | 6.64E-02 | brown4 |

| | | | | | | | |
|---|---|---|---|---|---|---|---|
| ENSG00000079482 | OPHN1 | 0.12 | 1.09 | 0.04 | 0.00091236 | 6.67E-02 | black |
| ENSG00000164211 | STARD4 | -0.15 | 0.90 | 0.05 | 0.00092152 | 6.70E-02 | brown |
| ENSG00000010319 | SEMA3G | -0.19 | 0.88 | 0.06 | 0.00094102 | 6.79E-02 | brown |
| ENSG00000143036 | SLC44A3 | 0.19 | 1.14 | 0.06 | 0.00094341 | 6.79E-02 | brown4 |
| ENSG00000081853 | PCDHGA2 | 0.17 | 1.12 | 0.05 | 0.00095464 | 6.84E-02 | black |
| ENSG00000149131 | SERPING1 | 0.18 | 1.13 | 0.05 | 0.00096289 | 6.87E-02 | brown4 |
| ENSG00000107242 | PIP5K1B | -0.15 | 0.90 | 0.05 | 0.00097279 | 6.90E-02 | mediumpurple3 |
| ENSG00000100949 | RABGGTA | -0.13 | 0.92 | 0.04 | 0.00099045 | 6.98E-02 | grey60 |
| ENSG00000107738 | C10orf54 | 0.19 | 1.14 | 0.06 | 0.00099368 | 6.98E-02 | brown4 |
| ENSG00000147121 | KRBOX4 | 0.11 | 1.08 | 0.03 | 0.00101508 | 7.07E-02 | brown4 |
| ENSG00000163492 | CCDC141 | -0.19 | 0.88 | 0.06 | 0.00101667 | 7.07E-02 | grey |
| ENSG00000175556 | LONRF3 | 0.18 | 1.13 | 0.05 | 0.0010352 | 7.17E-02 | brown4 |
| ENSG00000137198 | GMPR | 0.19 | 1.14 | 0.06 | 0.00105632 | 7.28E-02 | brown4 |
| ENSG00000197647 | ZNF433 | 0.14 | 1.11 | 0.04 | 0.00111355 | 7.64E-02 | brown4 |
| ENSG00000104899 | AMH | 0.19 | 1.14 | 0.06 | 0.00112179 | 7.64E-02 | yellow |
| ENSG00000135414 | GDF11 | -0.13 | 0.91 | 0.04 | 0.00113565 | 7.64E-02 | salmon4 |
| ENSG00000111328 | CDK2AP1 | -0.14 | 0.91 | 0.04 | 0.00113575 | 7.64E-02 | paleturquoise |
| ENSG00000257335 | MGAM | 0.18 | 1.14 | 0.06 | 0.00114104 | 7.64E-02 | brown4 |
| ENSG00000181396 | OGFOD3 | 0.11 | 1.08 | 0.03 | 0.00114453 | 7.64E-02 | tan |
| ENSG00000214456 | PLIN5 | 0.19 | 1.14 | 0.06 | 0.00114558 | 7.64E-02 | brown4 |
| ENSG00000130208 | APOC1 | -0.19 | 0.88 | 0.06 | 0.00117315 | 7.72E-02 | lightcyan |
| ENSG00000131969 | ABHD12B | -0.13 | 0.91 | 0.04 | 0.00117352 | 7.72E-02 | lightsteelblue1 |
| ENSG00000104365 | IKBKB | -0.10 | 0.93 | 0.03 | 0.00117353 | 7.72E-02 | blue |
| ENSG00000047457 | CP | 0.16 | 1.11 | 0.05 | 0.00118794 | 7.77E-02 | brown4 |
| ENSG00000151632 | AKR1C2 | 0.18 | 1.13 | 0.06 | 0.00120524 | 7.77E-02 | grey |
| ENSG00000148498 | PARD3 | 0.17 | 1.12 | 0.05 | 0.00121211 | 7.77E-02 | black |
| ENSG00000100156 | SLC16A8 | 0.18 | 1.13 | 0.06 | 0.00121251 | 7.77E-02 | plum1 |
| ENSG00000171246 | NPTX1 | 0.15 | 1.11 | 0.05 | 0.00121741 | 7.77E-02 | darkorange2 |
| ENSG00000170325 | PRDM10 | -0.08 | 0.94 | 0.03 | 0.00122228 | 7.77E-02 | lightsteelblue1 |
| ENSG00000162419 | GMEB1 | -0.09 | 0.94 | 0.03 | 0.00122302 | 7.77E-02 | darkolivegreen |
| ENSG00000221968 | FADS3 | 0.12 | 1.09 | 0.04 | 0.00122323 | 7.77E-02 | yellow |
| ENSG00000140798 | ABCC12 | -0.17 | 0.89 | 0.05 | 0.00124488 | 7.87E-02 | brown |
| ENSG00000170439 | METTL7B | 0.19 | 1.14 | 0.06 | 0.00125739 | 7.91E-02 | brown4 |
| ENSG00000197747 | S100A10 | 0.19 | 1.14 | 0.06 | 0.00128206 | 8.03E-02 | brown4 |
| ENSG00000104823 | ECH1 | 0.11 | 1.08 | 0.04 | 0.00128722 | 8.03E-02 | darkolivegreen |
| ENSG00000168874 | ATOH8 | 0.19 | 1.14 | 0.06 | 0.00130202 | 8.03E-02 | brown4 |
| ENSG00000145832 | SLC25A48 | 0.19 | 1.14 | 0.06 | 0.00130498 | 8.03E-02 | blue |
| ENSG00000168394 | TAP1 | 0.18 | 1.13 | 0.05 | 0.00130856 | 8.03E-02 | palevioletred3 |
| ENSG00000175899 | A2M | -0.17 | 0.89 | 0.05 | 0.00130988 | 8.03E-02 | lightcyan |
| ENSG00000165478 | HEPACAM | 0.17 | 1.12 | 0.05 | 0.001326 | 8.05E-02 | brown4 |
| ENSG00000137822 | TUBGCP4 | -0.07 | 0.95 | 0.02 | 0.00132828 | 8.05E-02 | lightsteelblue1 |
| ENSG00000176907 | C8orf4 | -0.18 | 0.88 | 0.06 | 0.00132911 | 8.05E-02 | brown |
| ENSG00000105127 | AKAP8 | 0.11 | 1.08 | 0.03 | 0.00134492 | 8.11E-02 | brown |
| ENSG00000097046 | CDC7 | -0.13 | 0.92 | 0.04 | 0.00135089 | 8.11E-02 | brown |
| ENSG00000108018 | SORCS1 | 0.10 | 1.07 | 0.03 | 0.00137696 | 8.24E-02 | lightsteelblue1 |
| ENSG00000105974 | CAV1 | -0.18 | 0.88 | 0.06 | 0.00138685 | 8.26E-02 | lightgreen |
| ENSG00000254245 | PCDHGA3 | 0.16 | 1.12 | 0.05 | 0.00140754 | 8.30E-02 | black |
| ENSG00000187608 | ISG15 | 0.18 | 1.13 | 0.06 | 0.00140831 | 8.30E-02 | palevioletred3 |
| ENSG00000102265 | TIMP1 | 0.19 | 1.14 | 0.06 | 0.00141406 | 8.30E-02 | brown4 |
| ENSG00000137513 | NARS2 | -0.12 | 0.92 | 0.04 | 0.00141562 | 8.30E-02 | brown |

| | | | | | | | |
|---|---|---|---|---|---|---|---|
| ENSG00000088280 | ASAP3 | 0.15 | 1.11 | 0.05 | 0.00143135 | 8.35E-02 | brown4 |
| ENSG00000174130 | TLR6 | -0.18 | 0.88 | 0.06 | 0.00145278 | 8.44E-02 | lightcyan |
| ENSG00000169738 | DCXR | 0.13 | 1.09 | 0.04 | 0.00148872 | 8.61E-02 | darkolivegreen |
| ENSG00000101333 | PLCB4 | -0.10 | 0.93 | 0.03 | 0.0015014 | 8.61E-02 | lightsteelblue1 |
| ENSG00000147571 | CRH | -0.18 | 0.88 | 0.06 | 0.00150149 | 8.61E-02 | darkslateblue |
| ENSG00000100243 | CYB5R3 | 0.12 | 1.09 | 0.04 | 0.00150727 | 8.61E-02 | brown4 |
| ENSG00000186026 | ZNF284 | -0.12 | 0.92 | 0.04 | 0.00151101 | 8.61E-02 | brown |
| ENSG00000160087 | UBE2J2 | 0.09 | 1.07 | 0.03 | 0.00152491 | 8.62E-02 | grey60 |
| ENSG00000113319 | RASGRF2 | -0.13 | 0.92 | 0.04 | 0.00152549 | 8.62E-02 | mediumpurple3 |
| ENSG00000101213 | PTK6 | 0.18 | 1.14 | 0.06 | 0.00154169 | 8.64E-02 | blue |
| ENSG00000121417 | ZNF211 | -0.08 | 0.95 | 0.03 | 0.00154492 | 8.64E-02 | paleturquoise |
| ENSG00000141665 | FBXO15 | -0.13 | 0.91 | 0.04 | 0.00154553 | 8.64E-02 | blue |
| ENSG00000144596 | GRIP2 | -0.14 | 0.91 | 0.05 | 0.00156597 | 8.72E-02 | grey60 |
| ENSG00000132274 | TRIM22 | 0.17 | 1.13 | 0.05 | 0.00158204 | 8.77E-02 | palevioletred3 |
| ENSG00000133687 | TMTC1 | 0.12 | 1.08 | 0.04 | 0.00161506 | 8.90E-02 | brown |
| ENSG00000180914 | OXTR | 0.18 | 1.13 | 0.06 | 0.00161686 | 8.90E-02 | brown4 |
| ENSG00000130589 | HELZ2 | 0.18 | 1.14 | 0.06 | 0.0016274 | 8.92E-02 | palevioletred3 |
| ENSG00000176014 | TUBB6 | 0.18 | 1.13 | 0.06 | 0.00164682 | 8.97E-02 | brown4 |
| ENSG00000125534 | PPDPF | 0.16 | 1.12 | 0.05 | 0.00164937 | 8.97E-02 | yellow |
| ENSG00000185245 | GP1BA | 0.17 | 1.13 | 0.06 | 0.00166359 | 9.01E-02 | yellow |
| ENSG00000165621 | OXGR1 | -0.18 | 0.88 | 0.06 | 0.00167726 | 9.01E-02 | mediumpurple3 |
| ENSG00000166979 | EVA1C | 0.13 | 1.09 | 0.04 | 0.00167846 | 9.01E-02 | blue |
| ENSG00000149599 | DUSP15 | 0.17 | 1.13 | 0.06 | 0.00168078 | 9.01E-02 | brown4 |
| ENSG00000182601 | HS3ST4 | 0.17 | 1.13 | 0.05 | 0.00170837 | 9.12E-02 | darkorange2 |
| ENSG00000076248 | UNG | 0.14 | 1.11 | 0.05 | 0.00172594 | 9.12E-02 | brown4 |
| ENSG00000128989 | ARPP19 | -0.13 | 0.92 | 0.04 | 0.00172909 | 9.12E-02 | brown |
| ENSG00000115616 | SLC9A2 | -0.16 | 0.89 | 0.05 | 0.0017322 | 9.12E-02 | brown |
| ENSG00000154975 | CA10 | -0.13 | 0.92 | 0.04 | 0.001733 | 9.12E-02 | mediumpurple3 |
| ENSG00000124787 | RPP40 | -0.14 | 0.91 | 0.04 | 0.0017393 | 9.12E-02 | brown |
| ENSG00000153902 | LGI4 | 0.14 | 1.10 | 0.04 | 0.00174447 | 9.12E-02 | brown4 |
| ENSG00000151466 | SCLT1 | -0.09 | 0.94 | 0.03 | 0.0017556 | 9.12E-02 | paleturquoise |
| ENSG00000152578 | GRIA4 | -0.12 | 0.92 | 0.04 | 0.00175726 | 9.12E-02 | mediumpurple3 |
| ENSG00000120903 | CHRNA2 | -0.17 | 0.89 | 0.06 | 0.00179842 | 9.26E-02 | thistle2 |
| ENSG00000163644 | PPM1K | 0.09 | 1.07 | 0.03 | 0.00179849 | 9.26E-02 | darkgrey |
| ENSG00000112576 | CCND3 | 0.11 | 1.08 | 0.04 | 0.00181863 | 9.33E-02 | brown4 |
| ENSG00000163312 | HELQ | -0.09 | 0.94 | 0.03 | 0.0018401 | 9.41E-02 | darkgrey |
| ENSG00000168918 | INPP5D | -0.16 | 0.89 | 0.05 | 0.0018521 | 9.42E-02 | lightcyan |
| ENSG00000243710 | WDR65 | -0.16 | 0.89 | 0.05 | 0.00185402 | 9.42E-02 | mediumpurple3 |
| ENSG00000008083 | JARID2 | 0.10 | 1.07 | 0.03 | 0.00186096 | 9.42E-02 | brown4 |
| ENSG00000143248 | RGS5 | -0.15 | 0.90 | 0.05 | 0.00186963 | 9.43E-02 | brown |
| ENSG00000185201 | IFITM2 | 0.17 | 1.13 | 0.06 | 0.00189951 | 9.51E-02 | brown4 |
| ENSG00000164089 | ETNPPL | 0.18 | 1.13 | 0.06 | 0.00190999 | 9.51E-02 | black |
| ENSG00000173376 | NDNF | -0.18 | 0.88 | 0.06 | 0.00191222 | 9.51E-02 | thistle2 |
| ENSG00000162927 | PUS10 | -0.11 | 0.92 | 0.04 | 0.00191291 | 9.51E-02 | turquoise |
| ENSG00000155380 | SLC16A1 | 0.18 | 1.13 | 0.06 | 0.00197997 | 9.81E-02 | brown4 |
| ENSG00000138678 | AGPAT9 | -0.16 | 0.90 | 0.05 | 0.0019858 | 9.81E-02 | brown |
| ENSG00000108932 | SLC16A6 | 0.17 | 1.12 | 0.05 | 0.00200673 | 9.88E-02 | brown4 |
| ENSG00000162738 | VANGL2 | 0.16 | 1.12 | 0.05 | 0.0020137 | 9.88E-02 | brown4 |
| ENSG00000188321 | ZNF559 | -0.12 | 0.92 | 0.04 | 0.00202472 | 9.90E-02 | paleturquoise |
| ENSG00000173588 | CCDC41 | -0.09 | 0.94 | 0.03 | 0.0020475 | 9.98E-02 | darkgrey |

| | | | | | | | |
|---|---|---|---|---|---|---|---|
| ENSG00000177030 | DEAF1 | 0.11 | 1.08 | 0.04 | 0.00207308 | 1.01E-01 | darkolivegreen |
| ENSG00000163535 | SGOL2 | -0.13 | 0.92 | 0.04 | 0.00208636 | 1.01E-01 | darkgrey |
| ENSG00000067955 | CBFB | 0.16 | 1.12 | 0.05 | 0.00210551 | 1.02E-01 | paleturquoise |
| ENSG00000125148 | MT2A | 0.18 | 1.13 | 0.06 | 0.00212755 | 1.02E-01 | brown4 |
| ENSG00000167525 | PROCA1 | 0.15 | 1.11 | 0.05 | 0.00213653 | 1.02E-01 | brown4 |
| ENSG00000105438 | KDELR1 | 0.08 | 1.06 | 0.03 | 0.00213897 | 1.02E-01 | yellow |
| ENSG00000197948 | FCHSD1 | 0.13 | 1.09 | 0.04 | 0.00213991 | 1.02E-01 | tan |
| ENSG00000170837 | GPR27 | 0.13 | 1.09 | 0.04 | 0.0021499 | 1.02E-01 | grey |
| ENSG00000117650 | NEK2 | -0.18 | 0.88 | 0.06 | 0.0021714 | 1.03E-01 | brown |
| ENSG00000124762 | CDKN1A | 0.16 | 1.12 | 0.05 | 0.00218153 | 1.03E-01 | brown4 |
| ENSG00000144619 | CNTN4 | -0.10 | 0.93 | 0.03 | 0.00218839 | 1.03E-01 | lightsteelblue1 |
| ENSG00000085721 | RRN3 | 0.11 | 1.08 | 0.04 | 0.00221684 | 1.04E-01 | darkgrey |
| ENSG00000161904 | LEMD2 | 0.09 | 1.06 | 0.03 | 0.00221818 | 1.04E-01 | blue |
| ENSG00000196396 | PTPN1 | 0.08 | 1.06 | 0.03 | 0.00224809 | 1.04E-01 | brown4 |
| ENSG00000005108 | THSD7A | -0.14 | 0.90 | 0.05 | 0.00225442 | 1.04E-01 | thistle2 |
| ENSG00000152760 | TCTEX1D1 | -0.15 | 0.90 | 0.05 | 0.00225806 | 1.04E-01 | lightsteelblue1 |
| ENSG00000110013 | SIAE | -0.10 | 0.94 | 0.03 | 0.00225866 | 1.04E-01 | brown |
| ENSG00000072952 | MRVI1 | 0.17 | 1.13 | 0.06 | 0.00230335 | 1.06E-01 | brown4 |
| ENSG00000154928 | EPHB1 | -0.13 | 0.92 | 0.04 | 0.00232749 | 1.06E-01 | salmon4 |
| ENSG00000173208 | ABCD2 | -0.13 | 0.91 | 0.04 | 0.00233222 | 1.06E-01 | lightsteelblue1 |
| ENSG00000126882 | FAM78A | -0.13 | 0.92 | 0.04 | 0.00233362 | 1.06E-01 | grey60 |
| ENSG00000170324 | FRMPD2 | -0.17 | 0.89 | 0.06 | 0.00234065 | 1.06E-01 | brown |
| ENSG00000196549 | MME | -0.16 | 0.90 | 0.05 | 0.00234856 | 1.06E-01 | lightsteelblue1 |
| ENSG00000185689 | C6orf201 | 0.16 | 1.12 | 0.05 | 0.00235487 | 1.06E-01 | grey |
| ENSG00000267508 | ZNF285 | -0.11 | 0.93 | 0.04 | 0.00239591 | 1.08E-01 | blue |
| ENSG00000103811 | CTSH | -0.15 | 0.90 | 0.05 | 0.00240826 | 1.08E-01 | brown4 |
| ENSG00000138795 | LEF1 | -0.15 | 0.90 | 0.05 | 0.00243234 | 1.09E-01 | lightgreen |
| ENSG00000167703 | SLC43A2 | 0.14 | 1.10 | 0.05 | 0.00245771 | 1.09E-01 | brown4 |
| ENSG00000153767 | GTF2E1 | -0.10 | 0.93 | 0.03 | 0.00246085 | 1.09E-01 | brown |
| ENSG00000069535 | MAOB | 0.12 | 1.09 | 0.04 | 0.0024788 | 1.10E-01 | brown4 |
| ENSG00000101276 | SLC52A3 | 0.17 | 1.13 | 0.06 | 0.00251698 | 1.11E-01 | brown4 |
| ENSG00000173517 | PEAK1 | -0.07 | 0.95 | 0.02 | 0.00252325 | 1.11E-01 | yellow |
| ENSG00000164379 | FOXQ1 | -0.17 | 0.89 | 0.06 | 0.00256424 | 1.12E-01 | grey |
| ENSG00000122254 | HS3ST2 | 0.18 | 1.13 | 0.06 | 0.00258127 | 1.13E-01 | darkorange2 |
| ENSG00000184304 | PRKD1 | -0.09 | 0.94 | 0.03 | 0.00259097 | 1.13E-01 | turquoise |
| ENSG00000182287 | AP1S2 | -0.12 | 0.92 | 0.04 | 0.00261367 | 1.14E-01 | brown |
| ENSG00000241563 | CORT | -0.17 | 0.89 | 0.06 | 0.00265815 | 1.15E-01 | grey60 |
| ENSG00000171488 | LRRC8C | -0.11 | 0.93 | 0.04 | 0.00268519 | 1.16E-01 | brown |
| ENSG00000106034 | CPED1 | -0.17 | 0.89 | 0.06 | 0.00271139 | 1.16E-01 | lightgreen |
| ENSG00000156049 | GNA14 | 0.16 | 1.11 | 0.05 | 0.00271461 | 1.16E-01 | brown4 |
| ENSG00000197081 | IGF2R | 0.07 | 1.05 | 0.02 | 0.00271841 | 1.16E-01 | yellow |
| ENSG00000162694 | EXTL2 | -0.13 | 0.92 | 0.04 | 0.00272285 | 1.16E-01 | lightsteelblue1 |
| ENSG00000136425 | CIB2 | 0.15 | 1.11 | 0.05 | 0.00273476 | 1.16E-01 | darkolivegreen |
| ENSG00000167100 | SAMD14 | 0.14 | 1.10 | 0.05 | 0.00275646 | 1.17E-01 | yellow |
| ENSG00000197299 | BLM | -0.13 | 0.91 | 0.04 | 0.00277656 | 1.18E-01 | brown |
| ENSG00000164326 | CARTPT | 0.15 | 1.11 | 0.05 | 0.00278558 | 1.18E-01 | lightsteelblue1 |
| ENSG00000197956 | S100A6 | 0.14 | 1.10 | 0.05 | 0.00280892 | 1.18E-01 | skyblue3 |
| ENSG00000181631 | P2RY13 | -0.15 | 0.90 | 0.05 | 0.00283036 | 1.18E-01 | lightcyan |
| ENSG00000167291 | TBC1D16 | 0.09 | 1.06 | 0.03 | 0.00284098 | 1.18E-01 | brown4 |
| ENSG00000169064 | ZBBX | -0.15 | 0.90 | 0.05 | 0.00284437 | 1.18E-01 | lightsteelblue1 |

| | | | | | | | |
|---|---|---|---|---|---|---|---|
| ENSG00000112280 | COL9A1 | -0.14 | 0.91 | 0.05 | 0.00284929 | 1.18E-01 | paleturquoise |
| ENSG00000095585 | BLNK | -0.17 | 0.89 | 0.06 | 0.00285023 | 1.18E-01 | lightcyan |
| ENSG00000172059 | KLF11 | 0.13 | 1.09 | 0.04 | 0.00286074 | 1.18E-01 | brown4 |
| ENSG00000203326 | ZNF525 | -0.13 | 0.91 | 0.04 | 0.00288338 | 1.19E-01 | paleturquoise |
| ENSG00000138821 | SLC39A8 | -0.13 | 0.91 | 0.04 | 0.00289384 | 1.19E-01 | darkgrey |
| ENSG00000116679 | IVNS1ABP | -0.13 | 0.92 | 0.04 | 0.00291385 | 1.19E-01 | paleturquoise |
| ENSG00000175054 | ATR | -0.07 | 0.95 | 0.02 | 0.00291785 | 1.19E-01 | brown |
| ENSG00000017483 | SLC38A5 | -0.17 | 0.89 | 0.06 | 0.00292026 | 1.19E-01 | brown |
| ENSG00000105122 | RASAL3 | -0.17 | 0.89 | 0.06 | 0.00293611 | 1.19E-01 | lightcyan |
| ENSG00000152034 | MCHR2 | -0.14 | 0.90 | 0.05 | 0.0029386 | 1.19E-01 | lightsteelblue1 |
| ENSG00000130529 | TRPM4 | 0.14 | 1.10 | 0.05 | 0.0029446 | 1.19E-01 | yellow |
| ENSG00000139352 | ASCL1 | -0.14 | 0.90 | 0.05 | 0.00296043 | 1.19E-01 | salmon4 |
| ENSG00000168491 | CCDC110 | -0.14 | 0.91 | 0.05 | 0.00297982 | 1.20E-01 | lightsteelblue1 |
| ENSG00000184270 | HIST2H2AB | 0.13 | 1.10 | 0.04 | 0.00298228 | 1.20E-01 | black |
| ENSG00000152595 | MEPE | 0.17 | 1.13 | 0.06 | 0.00299615 | 1.20E-01 | brown |
| ENSG00000143772 | ITPKB | 0.16 | 1.12 | 0.05 | 0.00300677 | 1.20E-01 | brown4 |
| ENSG00000138669 | PRKG2 | -0.12 | 0.92 | 0.04 | 0.00305596 | 1.21E-01 | mediumpurple3 |
| ENSG00000177951 | BET1L | 0.09 | 1.06 | 0.03 | 0.0030562 | 1.21E-01 | yellow |
| ENSG00000101349 | PAK7 | -0.10 | 0.93 | 0.03 | 0.00307406 | 1.21E-01 | lightsteelblue1 |
| ENSG00000115415 | STAT1 | 0.15 | 1.11 | 0.05 | 0.00308019 | 1.21E-01 | palevioletred3 |
| ENSG00000106351 | AGFG2 | 0.09 | 1.06 | 0.03 | 0.00308676 | 1.21E-01 | brown4 |
| ENSG00000066382 | MPPED2 | -0.09 | 0.94 | 0.03 | 0.00310572 | 1.22E-01 | mediumpurple3 |
| ENSG00000102393 | GLA | -0.14 | 0.91 | 0.05 | 0.00310693 | 1.22E-01 | brown |
| ENSG00000116729 | WLS | 0.13 | 1.09 | 0.04 | 0.00312755 | 1.22E-01 | brown4 |
| ENSG00000166949 | SMAD3 | -0.09 | 0.94 | 0.03 | 0.00314068 | 1.22E-01 | grey60 |
| ENSG00000115239 | GPR75-ASB3 | 0.09 | 1.07 | 0.03 | 0.00314394 | 1.22E-01 | black |
| ENSG00000160124 | CCDC58 | -0.11 | 0.93 | 0.04 | 0.00317414 | 1.23E-01 | darkgrey |
| ENSG00000175077 | RTP1 | -0.17 | 0.89 | 0.06 | 0.00318938 | 1.23E-01 | lightsteelblue1 |
| ENSG00000139410 | SDSL | 0.17 | 1.13 | 0.06 | 0.00322642 | 1.24E-01 | brown4 |
| ENSG00000145451 | GLRA3 | -0.14 | 0.91 | 0.05 | 0.00324943 | 1.25E-01 | mediumpurple3 |
| ENSG00000122547 | EEPD1 | -0.12 | 0.92 | 0.04 | 0.00329801 | 1.25E-01 | blue |
| ENSG00000021461 | CYP3A43 | 0.17 | 1.12 | 0.06 | 0.0033114 | 1.25E-01 | turquoise |
| ENSG00000173926 | 43162 | 0.17 | 1.12 | 0.06 | 0.00331468 | 1.25E-01 | brown4 |
| ENSG00000197019 | SERTAD1 | 0.17 | 1.13 | 0.06 | 0.00332166 | 1.25E-01 | plum2 |
| ENSG00000118432 | CNR1 | -0.13 | 0.91 | 0.04 | 0.00333094 | 1.25E-01 | thistle2 |
| ENSG00000127252 | HRASLS | -0.12 | 0.92 | 0.04 | 0.00333158 | 1.25E-01 | brown |
| ENSG00000109111 | SUPT6H | 0.08 | 1.06 | 0.03 | 0.00333306 | 1.25E-01 | yellow |
| ENSG00000168003 | SLC3A2 | 0.12 | 1.09 | 0.04 | 0.00333396 | 1.25E-01 | brown4 |
| ENSG00000125520 | SLC2A4RG | 0.14 | 1.10 | 0.05 | 0.00335437 | 1.26E-01 | brown4 |
| ENSG00000133048 | CHI3L1 | 0.14 | 1.10 | 0.05 | 0.00336823 | 1.26E-01 | brown4 |
| ENSG00000056277 | ZNF280C | -0.08 | 0.94 | 0.03 | 0.00338503 | 1.26E-01 | darkgrey |
| ENSG00000185885 | IFITM1 | 0.17 | 1.12 | 0.06 | 0.00339451 | 1.26E-01 | palevioletred3 |
| ENSG00000005243 | COPZ2 | 0.16 | 1.12 | 0.05 | 0.00340792 | 1.26E-01 | brown4 |
| ENSG00000143367 | TUFT1 | 0.10 | 1.07 | 0.03 | 0.00342974 | 1.27E-01 | grey60 |
| ENSG00000174371 | EXO1 | -0.16 | 0.90 | 0.05 | 0.00343029 | 1.27E-01 | thistle2 |
| ENSG00000141485 | SLC13A5 | -0.16 | 0.89 | 0.06 | 0.00344228 | 1.27E-01 | salmon4 |
| ENSG00000183682 | BMP8A | 0.13 | 1.10 | 0.05 | 0.00344974 | 1.27E-01 | grey60 |
| ENSG00000165970 | SLC6A5 | -0.16 | 0.89 | 0.05 | 0.00345607 | 1.27E-01 | lightsteelblue1 |
| ENSG00000133710 | SPINK5 | -0.14 | 0.91 | 0.05 | 0.00351244 | 1.28E-01 | blue |
| ENSG00000141337 | ARSG | 0.12 | 1.09 | 0.04 | 0.00353909 | 1.28E-01 | tan |

| | | | | | | | |
|---|---|---|---|---|---|---|---|
| ENSG00000224470 | ATXN1L | 0.08 | 1.05 | 0.03 | 0.00354065 | 1.28E-01 | blue |
| ENSG00000088992 | TESC | -0.14 | 0.91 | 0.05 | 0.00354618 | 1.28E-01 | darkolivegreen |
| ENSG00000211448 | DIO2 | -0.17 | 0.89 | 0.06 | 0.00355042 | 1.28E-01 | black |
| ENSG00000160224 | AIRE | 0.17 | 1.12 | 0.06 | 0.00355426 | 1.28E-01 | yellow |
| ENSG00000156535 | CD109 | 0.14 | 1.11 | 0.05 | 0.0035554 | 1.28E-01 | black |
| ENSG00000159403 | C1R | 0.17 | 1.13 | 0.06 | 0.00356552 | 1.28E-01 | brown4 |
| ENSG00000128928 | IVD | 0.08 | 1.06 | 0.03 | 0.00357711 | 1.28E-01 | brown4 |
| ENSG00000130940 | CASZ1 | 0.16 | 1.12 | 0.06 | 0.0035854 | 1.28E-01 | brown4 |
| ENSG00000198721 | ECI2 | 0.12 | 1.09 | 0.04 | 0.00359795 | 1.28E-01 | brown4 |
| ENSG00000128606 | LRRC17 | -0.15 | 0.90 | 0.05 | 0.00362264 | 1.29E-01 | grey |
| ENSG00000137720 | C11orf1 | -0.10 | 0.93 | 0.03 | 0.00365851 | 1.30E-01 | brown |
| ENSG00000072682 | P4HA2 | 0.11 | 1.08 | 0.04 | 0.00368205 | 1.30E-01 | yellow |
| ENSG00000205639 | MFSD2B | 0.16 | 1.12 | 0.06 | 0.00369026 | 1.30E-01 | turquoise |
| ENSG00000115604 | IL18R1 | 0.14 | 1.10 | 0.05 | 0.00369955 | 1.30E-01 | brown4 |
| ENSG00000176293 | ZNF135 | -0.10 | 0.93 | 0.04 | 0.00370283 | 1.30E-01 | blue |
| ENSG00000010810 | FYN | 0.09 | 1.07 | 0.03 | 0.003718 | 1.30E-01 | brown4 |
| ENSG00000164344 | KLKB1 | -0.16 | 0.89 | 0.06 | 0.00374033 | 1.31E-01 | black |
| ENSG00000166292 | TMEM100 | 0.17 | 1.12 | 0.06 | 0.00382403 | 1.33E-01 | black |
| ENSG00000172243 | CLEC7A | -0.17 | 0.89 | 0.06 | 0.00385658 | 1.34E-01 | lightcyan |
| ENSG00000140678 | ITGAX | -0.17 | 0.89 | 0.06 | 0.00386303 | 1.34E-01 | lightcyan |
| ENSG00000180881 | CAPS2 | -0.10 | 0.93 | 0.04 | 0.0038725 | 1.34E-01 | lightsteelblue1 |
| ENSG00000241852 | C8orf58 | 0.13 | 1.10 | 0.05 | 0.00390332 | 1.35E-01 | brown4 |
| ENSG00000183943 | PRKX | 0.14 | 1.11 | 0.05 | 0.00390644 | 1.35E-01 | brown4 |
| ENSG00000165449 | SLC16A9 | 0.17 | 1.12 | 0.06 | 0.00392791 | 1.35E-01 | black |
| ENSG00000042493 | CAPG | 0.17 | 1.12 | 0.06 | 0.00393882 | 1.35E-01 | brown4 |
| ENSG00000072210 | ALDH3A2 | 0.09 | 1.07 | 0.03 | 0.0039469 | 1.35E-01 | darkgrey |
| ENSG00000110628 | SLC22A18 | 0.16 | 1.11 | 0.05 | 0.00395235 | 1.35E-01 | grey60 |
| ENSG00000156017 | C9orf41 | -0.10 | 0.93 | 0.04 | 0.00396488 | 1.35E-01 | lightsteelblue1 |
| ENSG00000107186 | MPDZ | -0.08 | 0.95 | 0.03 | 0.00396747 | 1.35E-01 | paleturquoise |
| ENSG00000175970 | UNC119B | 0.07 | 1.05 | 0.02 | 0.00397307 | 1.35E-01 | grey |
| ENSG00000128655 | PDE11A | -0.16 | 0.90 | 0.05 | 0.00397858 | 1.35E-01 | blue |
| ENSG00000156140 | ADAMTS3 | -0.13 | 0.92 | 0.04 | 0.00403519 | 1.36E-01 | mediumpurple3 |
| ENSG00000147082 | CCNB3 | -0.16 | 0.89 | 0.06 | 0.00406424 | 1.37E-01 | blue |
| ENSG00000070269 | TMEM260 | -0.08 | 0.95 | 0.03 | 0.00407812 | 1.37E-01 | paleturquoise |
| ENSG00000072182 | ASIC4 | -0.14 | 0.91 | 0.05 | 0.00408773 | 1.37E-01 | grey60 |
| ENSG00000179918 | SEPHS2 | 0.11 | 1.08 | 0.04 | 0.004099 | 1.37E-01 | grey60 |
| ENSG00000166228 | PCBD1 | 0.11 | 1.08 | 0.04 | 0.00410234 | 1.37E-01 | brown4 |
| ENSG00000185507 | IRF7 | 0.15 | 1.11 | 0.05 | 0.00412963 | 1.37E-01 | brown4 |
| ENSG00000146469 | VIP | -0.16 | 0.89 | 0.06 | 0.0041625 | 1.38E-01 | thistle2 |
| ENSG00000100439 | ABHD4 | 0.15 | 1.11 | 0.05 | 0.00418023 | 1.38E-01 | brown4 |
| ENSG00000013503 | POLR3B | -0.08 | 0.95 | 0.03 | 0.0041923 | 1.38E-01 | brown |
| ENSG00000119737 | GPR75 | 0.13 | 1.09 | 0.04 | 0.00419468 | 1.38E-01 | black |
| ENSG00000087008 | ACOX3 | 0.07 | 1.05 | 0.03 | 0.00421104 | 1.39E-01 | yellow |
| ENSG00000026508 | CD44 | 0.14 | 1.11 | 0.05 | 0.00425293 | 1.39E-01 | brown4 |
| ENSG00000197093 | GAL3ST4 | -0.16 | 0.90 | 0.06 | 0.00425913 | 1.39E-01 | lightcyan |
| ENSG00000178623 | GPR35 | 0.16 | 1.11 | 0.05 | 0.00426172 | 1.39E-01 | yellow |
| ENSG00000183570 | PCBP3 | 0.13 | 1.09 | 0.04 | 0.00427767 | 1.39E-01 | yellow |
| ENSG00000115750 | TAF1B | -0.08 | 0.94 | 0.03 | 0.00435669 | 1.42E-01 | brown |
| ENSG00000121060 | TRIM25 | 0.12 | 1.09 | 0.04 | 0.0043868 | 1.42E-01 | palevioletred3 |
| ENSG00000170412 | GPRC5C | 0.16 | 1.12 | 0.06 | 0.00442527 | 1.43E-01 | brown4 |

| | | | | | | | |
|---|---|---|---|---|---|---|---|
| ENSG00000197037 | ZSCAN25 | 0.06 | 1.04 | 0.02 | 0.00445771 | 1.44E-01 | tan |
| ENSG00000116985 | BMP8B | 0.13 | 1.09 | 0.04 | 0.00447614 | 1.44E-01 | blue |
| ENSG00000101825 | MXRA5 | -0.16 | 0.89 | 0.06 | 0.00448647 | 1.44E-01 | brown |
| ENSG00000081320 | STK17B | 0.17 | 1.12 | 0.06 | 0.00455348 | 1.46E-01 | black |
| ENSG00000038532 | CLEC16A | 0.06 | 1.04 | 0.02 | 0.00458524 | 1.47E-01 | yellow |
| ENSG00000141434 | MEP1B | 0.15 | 1.11 | 0.05 | 0.00459106 | 1.47E-01 | turquoise |
| ENSG00000214050 | FBXO16 | -0.10 | 0.93 | 0.04 | 0.00461562 | 1.47E-01 | lightsteelblue1 |
| ENSG00000186480 | INSIG1 | -0.14 | 0.91 | 0.05 | 0.00465018 | 1.48E-01 | brown |
| ENSG00000120675 | DNAJC15 | -0.12 | 0.92 | 0.04 | 0.00465322 | 1.48E-01 | paleturquoise |
| ENSG00000134874 | DZIP1 | 0.08 | 1.06 | 0.03 | 0.00471675 | 1.49E-01 | yellow |
| ENSG00000070366 | SMG6 | 0.08 | 1.06 | 0.03 | 0.00476131 | 1.50E-01 | brown4 |
| ENSG00000118729 | CASQ2 | -0.15 | 0.90 | 0.05 | 0.00479059 | 1.51E-01 | lightgreen |
| ENSG00000189114 | BLOC1S3 | 0.11 | 1.08 | 0.04 | 0.00483294 | 1.51E-01 | yellow |
| ENSG00000182324 | KCNJ14 | -0.15 | 0.90 | 0.05 | 0.00483801 | 1.51E-01 | grey60 |
| ENSG00000187123 | LYPD6 | -0.12 | 0.92 | 0.04 | 0.00483891 | 1.51E-01 | thistle2 |
| ENSG00000198053 | SIRPA | 0.09 | 1.06 | 0.03 | 0.00484677 | 1.51E-01 | yellow |
| ENSG00000185950 | IRS2 | 0.10 | 1.07 | 0.04 | 0.00484936 | 1.51E-01 | yellow |
| ENSG00000070882 | OSBPL3 | -0.11 | 0.93 | 0.04 | 0.00495057 | 1.54E-01 | brown |
| ENSG00000198298 | ZNF485 | -0.12 | 0.92 | 0.04 | 0.00496242 | 1.54E-01 | paleturquoise |
| ENSG00000177707 | PVRL3 | -0.12 | 0.92 | 0.04 | 0.00499433 | 1.54E-01 | mediumpurple3 |
| ENSG00000197016 | ZNF470 | -0.09 | 0.94 | 0.03 | 0.00500381 | 1.54E-01 | brown |
| ENSG00000106633 | GCK | 0.14 | 1.10 | 0.05 | 0.00501058 | 1.54E-01 | darkolivegreen |
| ENSG00000146143 | PRIM2 | -0.10 | 0.94 | 0.03 | 0.00501611 | 1.54E-01 | paleturquoise |
| ENSG00000100226 | GTPBP1 | 0.08 | 1.06 | 0.03 | 0.00502229 | 1.54E-01 | darkolivegreen |
| ENSG00000147124 | ZNF41 | -0.07 | 0.95 | 0.03 | 0.00503034 | 1.54E-01 | brown |
| ENSG00000146242 | TPBG | -0.15 | 0.90 | 0.05 | 0.00504203 | 1.54E-01 | lightsteelblue1 |
| ENSG00000103319 | EEF2K | 0.10 | 1.07 | 0.04 | 0.00505105 | 1.54E-01 | brown4 |
| ENSG00000204611 | ZNF616 | -0.09 | 0.94 | 0.03 | 0.00505747 | 1.54E-01 | paleturquoise |
| ENSG00000181220 | ZNF746 | 0.08 | 1.06 | 0.03 | 0.00508174 | 1.54E-01 | yellow |
| ENSG00000118596 | SLC16A7 | -0.11 | 0.93 | 0.04 | 0.00508405 | 1.54E-01 | lightsteelblue1 |
| ENSG00000169129 | AFAP1L2 | -0.10 | 0.93 | 0.04 | 0.00514743 | 1.56E-01 | grey60 |
| ENSG00000160801 | PTH1R | 0.14 | 1.10 | 0.05 | 0.00519553 | 1.57E-01 | brown4 |
| ENSG00000067182 | TNFRSF1A | 0.16 | 1.11 | 0.06 | 0.00520479 | 1.57E-01 | brown4 |
| ENSG00000171160 | MORN4 | -0.09 | 0.94 | 0.03 | 0.00522373 | 1.57E-01 | grey60 |
| ENSG00000122971 | ACADS | 0.14 | 1.10 | 0.05 | 0.00522448 | 1.57E-01 | brown4 |
| ENSG00000168675 | LDLRAD4 | -0.10 | 0.93 | 0.03 | 0.00523331 | 1.57E-01 | paleturquoise |
| ENSG00000101104 | PABPC1L | -0.12 | 0.92 | 0.04 | 0.00524808 | 1.57E-01 | tan |
| ENSG00000169083 | AR | -0.13 | 0.91 | 0.05 | 0.0052488 | 1.57E-01 | darkgrey |
| ENSG00000143393 | PI4KB | 0.08 | 1.05 | 0.03 | 0.00528074 | 1.57E-01 | darkolivegreen |
| ENSG00000145248 | SLC10A4 | -0.15 | 0.90 | 0.05 | 0.00530817 | 1.57E-01 | thistle2 |
| ENSG00000004864 | SLC25A13 | -0.15 | 0.90 | 0.05 | 0.00531236 | 1.57E-01 | blue |
| ENSG00000136802 | LRRC8A | 0.11 | 1.08 | 0.04 | 0.00531312 | 1.57E-01 | black |
| ENSG00000162601 | MYSM1 | -0.08 | 0.94 | 0.03 | 0.00535535 | 1.58E-01 | lightsteelblue1 |
| ENSG00000018869 | ZNF582 | -0.08 | 0.95 | 0.03 | 0.00536496 | 1.58E-01 | paleturquoise |
| ENSG00000173391 | OLR1 | -0.16 | 0.90 | 0.06 | 0.00538233 | 1.58E-01 | lightcyan |
| ENSG00000131435 | PDLIM4 | 0.16 | 1.12 | 0.06 | 0.00538259 | 1.58E-01 | brown4 |
| ENSG00000181104 | F2R | 0.15 | 1.11 | 0.06 | 0.00538716 | 1.58E-01 | brown4 |
| ENSG00000164142 | FAM160A1 | -0.14 | 0.91 | 0.05 | 0.00540944 | 1.58E-01 | grey60 |
| ENSG00000140939 | NOL3 | 0.13 | 1.10 | 0.05 | 0.00544126 | 1.59E-01 | blue |
| ENSG00000205758 | CRYZL1 | 0.06 | 1.05 | 0.02 | 0.00545587 | 1.59E-01 | brown |

| | | | | | | | |
|---|---|---|---|---|---|---|---|
| ENSG00000176222 | ZNF404 | -0.11 | 0.93 | 0.04 | 0.00545902 | 1.59E-01 | paleturquoise |
| ENSG00000152495 | CAMK4 | -0.12 | 0.92 | 0.04 | 0.00554541 | 1.60E-01 | lightsteelblue1 |
| ENSG00000175445 | LPL | 0.15 | 1.11 | 0.06 | 0.00554709 | 1.60E-01 | darkgrey |
| ENSG00000196235 | SUPT5H | 0.08 | 1.06 | 0.03 | 0.00556093 | 1.60E-01 | darkolivegreen |
| ENSG00000136859 | ANGPTL2 | -0.14 | 0.91 | 0.05 | 0.0055653 | 1.60E-01 | blue |
| ENSG00000118292 | C1orf54 | 0.16 | 1.12 | 0.06 | 0.00557529 | 1.60E-01 | brown4 |
| ENSG00000182463 | TSHZ2 | 0.14 | 1.10 | 0.05 | 0.00559442 | 1.61E-01 | saddlebrown |
| ENSG00000179941 | BBS10 | -0.10 | 0.93 | 0.04 | 0.00563212 | 1.61E-01 | darkgrey |
| ENSG00000062598 | ELMO2 | 0.07 | 1.05 | 0.02 | 0.00565332 | 1.62E-01 | blue |
| ENSG00000117791 | 43161 | 0.09 | 1.07 | 0.03 | 0.00567705 | 1.62E-01 | brown4 |
| ENSG00000110975 | SYT10 | -0.15 | 0.90 | 0.05 | 0.00568346 | 1.62E-01 | lightsteelblue1 |
| ENSG00000128641 | MYO1B | -0.12 | 0.92 | 0.05 | 0.00574135 | 1.63E-01 | mediumpurple3 |
| ENSG00000134243 | SORT1 | 0.10 | 1.07 | 0.04 | 0.00577007 | 1.63E-01 | blue |
| ENSG00000157404 | KIT | -0.14 | 0.91 | 0.05 | 0.00577447 | 1.63E-01 | thistle2 |
| ENSG00000145725 | PPIP5K2 | -0.07 | 0.95 | 0.03 | 0.00579004 | 1.63E-01 | brown |
| ENSG00000117594 | HSD11B1 | -0.16 | 0.89 | 0.06 | 0.00579551 | 1.63E-01 | blue |
| ENSG00000005844 | ITGAL | -0.16 | 0.89 | 0.06 | 0.00579838 | 1.63E-01 | lightcyan |
| ENSG00000240583 | AQP1 | 0.14 | 1.10 | 0.05 | 0.00581995 | 1.64E-01 | blue |
| ENSG00000168792 | ABHD15 | 0.14 | 1.10 | 0.05 | 0.00583591 | 1.64E-01 | brown4 |
| ENSG00000077092 | RARB | -0.12 | 0.92 | 0.05 | 0.00584959 | 1.64E-01 | lightsteelblue1 |
| ENSG00000186395 | KRT10 | 0.11 | 1.08 | 0.04 | 0.00585285 | 1.64E-01 | yellowgreen |
| ENSG00000165061 | ZMAT4 | -0.12 | 0.92 | 0.04 | 0.0059029 | 1.64E-01 | lightsteelblue1 |
| ENSG00000151468 | CCDC3 | 0.10 | 1.07 | 0.04 | 0.00590525 | 1.64E-01 | darkolivegreen |
| ENSG00000075151 | EIF4G3 | -0.07 | 0.95 | 0.03 | 0.00591943 | 1.64E-01 | brown |
| ENSG00000186352 | ANKRD37 | -0.15 | 0.90 | 0.06 | 0.00605164 | 1.68E-01 | grey |
| ENSG00000077238 | IL4R | 0.16 | 1.11 | 0.06 | 0.00606261 | 1.68E-01 | brown4 |
| ENSG00000154764 | WNT7A | -0.14 | 0.91 | 0.05 | 0.00608173 | 1.68E-01 | thistle2 |
| ENSG00000174306 | ZHX3 | 0.08 | 1.05 | 0.03 | 0.00608933 | 1.68E-01 | black |
| ENSG00000119973 | PRLHR | -0.15 | 0.90 | 0.06 | 0.00616148 | 1.69E-01 | thistle2 |
| ENSG00000054277 | OPN3 | -0.13 | 0.92 | 0.05 | 0.0061794 | 1.69E-01 | brown |
| ENSG00000051620 | HEBP2 | 0.13 | 1.10 | 0.05 | 0.00618122 | 1.69E-01 | brown4 |
| ENSG00000214113 | LYRM4 | -0.08 | 0.94 | 0.03 | 0.00618425 | 1.69E-01 | brown |
| ENSG00000169313 | P2RY12 | -0.16 | 0.90 | 0.06 | 0.00618664 | 1.69E-01 | lightcyan |
| ENSG00000197921 | HES5 | -0.16 | 0.90 | 0.06 | 0.006214 | 1.69E-01 | salmon4 |
| ENSG00000109113 | RAB34 | 0.15 | 1.11 | 0.06 | 0.00626067 | 1.70E-01 | brown4 |
| ENSG00000104903 | LYL1 | -0.16 | 0.90 | 0.06 | 0.00631529 | 1.71E-01 | lightcyan |
| ENSG00000103196 | CRISPLD2 | 0.16 | 1.12 | 0.06 | 0.00631801 | 1.71E-01 | brown4 |
| ENSG00000088836 | SLC4A11 | 0.16 | 1.12 | 0.06 | 0.00633645 | 1.71E-01 | yellow |
| ENSG00000197520 | FAM177B | -0.16 | 0.90 | 0.06 | 0.00635681 | 1.71E-01 | lightcyan |
| ENSG00000165124 | SVEP1 | 0.16 | 1.12 | 0.06 | 0.00637567 | 1.71E-01 | blue |
| ENSG00000078401 | EDN1 | 0.15 | 1.11 | 0.06 | 0.00637642 | 1.71E-01 | brown4 |
| ENSG00000108091 | CCDC6 | 0.09 | 1.06 | 0.03 | 0.0063842 | 1.71E-01 | lightsteelblue1 |
| ENSG00000063761 | ADCK1 | -0.11 | 0.92 | 0.04 | 0.00639913 | 1.71E-01 | plum1 |
| ENSG00000143179 | UCK2 | -0.08 | 0.95 | 0.03 | 0.00640966 | 1.71E-01 | grey60 |
| ENSG00000142609 | C1orf222 | -0.13 | 0.91 | 0.05 | 0.00647576 | 1.73E-01 | yellow |
| ENSG00000198301 | SDAD1 | -0.08 | 0.95 | 0.03 | 0.00653278 | 1.74E-01 | brown |
| ENSG00000109906 | ZBTB16 | 0.13 | 1.09 | 0.05 | 0.00653609 | 1.74E-01 | yellow |
| ENSG00000196950 | SLC39A10 | -0.12 | 0.92 | 0.04 | 0.00655055 | 1.74E-01 | brown |
| ENSG00000005189 | AC004381.6 | -0.11 | 0.93 | 0.04 | 0.00657489 | 1.74E-01 | grey |
| ENSG00000135472 | FAIM2 | 0.10 | 1.07 | 0.04 | 0.00661914 | 1.75E-01 | grey60 |

| | | | | | | | |
|---|---|---|---|---|---|---|---|
| ENSG00000164292 | RHOBTB3 | 0.15 | 1.11 | 0.05 | 0.00663498 | 1.75E-01 | black |
| ENSG00000140519 | RHCG | 0.11 | 1.08 | 0.04 | 0.00667595 | 1.75E-01 | yellow |
| ENSG00000168958 | MFF | -0.08 | 0.95 | 0.03 | 0.00667713 | 1.75E-01 | paleturquoise |
| ENSG00000168309 | FAM107A | 0.15 | 1.11 | 0.05 | 0.00670648 | 1.75E-01 | brown4 |
| ENSG00000172824 | CES4A | 0.12 | 1.09 | 0.04 | 0.00670969 | 1.75E-01 | tan |
| ENSG00000177575 | CD163 | 0.12 | 1.09 | 0.04 | 0.00671875 | 1.75E-01 | brown4 |
| ENSG00000119685 | TTLL5 | -0.06 | 0.96 | 0.02 | 0.00672322 | 1.75E-01 | blue |
| ENSG00000066629 | EML1 | -0.07 | 0.95 | 0.03 | 0.00672356 | 1.75E-01 | paleturquoise |
| ENSG00000112312 | GMNN | 0.15 | 1.11 | 0.06 | 0.00673063 | 1.75E-01 | brown4 |
| ENSG00000175766 | EIF4E1B | -0.13 | 0.91 | 0.05 | 0.00674398 | 1.75E-01 | darkolivegreen |
| ENSG00000145623 | OSMR | 0.15 | 1.11 | 0.06 | 0.0067624 | 1.75E-01 | brown4 |
| ENSG00000187474 | FPR3 | 0.16 | 1.12 | 0.06 | 0.00677406 | 1.75E-01 | brown4 |
| ENSG00000081148 | IMPG2 | -0.12 | 0.92 | 0.05 | 0.0068105 | 1.75E-01 | lightsteelblue1 |
| ENSG00000076321 | KLHL20 | -0.08 | 0.95 | 0.03 | 0.00681248 | 1.75E-01 | paleturquoise |
| ENSG00000119917 | IFIT3 | 0.16 | 1.11 | 0.06 | 0.00681822 | 1.75E-01 | palevioletred3 |
| ENSG00000121578 | B4GALT4 | 0.08 | 1.06 | 0.03 | 0.0068248 | 1.75E-01 | brown4 |
| ENSG00000246705 | H2AFJ | 0.15 | 1.11 | 0.06 | 0.00683396 | 1.75E-01 | brown4 |
| ENSG00000147434 | CHRNA6 | -0.15 | 0.90 | 0.06 | 0.00683734 | 1.75E-01 | thistle2 |
| ENSG00000074181 | NOTCH3 | -0.15 | 0.90 | 0.06 | 0.00683868 | 1.75E-01 | lightgreen |
| ENSG00000135503 | ACVR1B | 0.08 | 1.06 | 0.03 | 0.00684896 | 1.75E-01 | grey60 |
| ENSG00000134326 | CMPK2 | 0.14 | 1.10 | 0.05 | 0.00685369 | 1.75E-01 | palevioletred3 |
| ENSG00000213088 | DARC | 0.16 | 1.12 | 0.06 | 0.00691078 | 1.76E-01 | brown4 |
| ENSG00000155324 | GRAMD3 | 0.14 | 1.11 | 0.05 | 0.00691519 | 1.76E-01 | black |
| ENSG00000112936 | C7 | 0.14 | 1.10 | 0.05 | 0.00692433 | 1.76E-01 | lightgreen |
| ENSG00000112319 | EYA4 | -0.14 | 0.91 | 0.05 | 0.00695681 | 1.76E-01 | paleturquoise |
| ENSG00000139687 | RB1 | -0.09 | 0.94 | 0.03 | 0.00696208 | 1.76E-01 | paleturquoise |
| ENSG00000186918 | ZNF395 | 0.11 | 1.08 | 0.04 | 0.00697179 | 1.76E-01 | brown4 |
| ENSG00000136250 | AOAH | -0.14 | 0.91 | 0.05 | 0.0069774 | 1.76E-01 | lightcyan |
| ENSG00000066427 | ATXN3 | -0.08 | 0.95 | 0.03 | 0.00701452 | 1.76E-01 | brown |
| ENSG00000118961 | C2orf43 | -0.10 | 0.93 | 0.04 | 0.00707222 | 1.77E-01 | brown |
| ENSG00000135108 | FBXO21 | 0.07 | 1.05 | 0.03 | 0.00708502 | 1.77E-01 | brown |
| ENSG00000103316 | CRYM | -0.13 | 0.91 | 0.05 | 0.00713187 | 1.78E-01 | darkolivegreen |
| ENSG00000213672 | NCKIPSD | 0.09 | 1.06 | 0.03 | 0.00714052 | 1.78E-01 | grey60 |
| ENSG00000197324 | LRP10 | 0.14 | 1.10 | 0.05 | 0.0071715 | 1.78E-01 | brown4 |
| ENSG00000127329 | PTPRB | -0.11 | 0.93 | 0.04 | 0.00717557 | 1.78E-01 | lightgreen |
| ENSG00000062096 | ARSF | -0.15 | 0.90 | 0.06 | 0.00721609 | 1.79E-01 | salmon4 |
| ENSG00000126010 | GRPR | -0.15 | 0.90 | 0.06 | 0.00721666 | 1.79E-01 | thistle2 |
| ENSG00000151789 | ZNF385D | -0.12 | 0.92 | 0.04 | 0.00726114 | 1.79E-01 | thistle2 |
| ENSG00000037280 | FLT4 | -0.15 | 0.90 | 0.05 | 0.00727606 | 1.79E-01 | darkolivegreen |
| ENSG00000157399 | ARSE | -0.13 | 0.91 | 0.05 | 0.00728951 | 1.79E-01 | grey |
| ENSG00000148516 | ZEB1 | -0.07 | 0.95 | 0.03 | 0.00729001 | 1.79E-01 | darkgrey |
| ENSG00000166821 | PEX11A | 0.11 | 1.08 | 0.04 | 0.00729323 | 1.79E-01 | black |
| ENSG00000105483 | CARD8 | -0.12 | 0.92 | 0.04 | 0.00730007 | 1.79E-01 | blue |
| ENSG00000075223 | SEMA3C | -0.14 | 0.91 | 0.05 | 0.00731752 | 1.79E-01 | paleturquoise |
| ENSG00000071243 | ING3 | -0.08 | 0.95 | 0.03 | 0.00736029 | 1.80E-01 | brown |
| ENSG00000165972 | CCDC38 | 0.15 | 1.11 | 0.06 | 0.00739211 | 1.80E-01 | turquoise |
| ENSG00000135547 | HEY2 | -0.14 | 0.91 | 0.05 | 0.00739494 | 1.80E-01 | lightgreen |
| ENSG00000126351 | THRA | 0.07 | 1.05 | 0.03 | 0.00747748 | 1.82E-01 | darkolivegreen |
| ENSG00000140443 | IGF1R | 0.08 | 1.06 | 0.03 | 0.00748751 | 1.82E-01 | yellow |
| ENSG00000153179 | RASSF3 | -0.12 | 0.92 | 0.05 | 0.00753554 | 1.83E-01 | brown |

| | | | | | | | |
|---|---|---|---|---|---|---|---|
| ENSG00000162595 | DIRAS3 | 0.14 | 1.10 | 0.05 | 0.00762286 | 1.84E-01 | brown |
| ENSG00000136205 | TNS3 | -0.13 | 0.91 | 0.05 | 0.00763082 | 1.84E-01 | salmon4 |
| ENSG00000198732 | SMOC1 | 0.15 | 1.11 | 0.06 | 0.00764348 | 1.84E-01 | blue |
| ENSG00000156787 | TBC1D31 | -0.08 | 0.94 | 0.03 | 0.00765592 | 1.84E-01 | paleturquoise |
| ENSG00000151164 | RAD9B | -0.13 | 0.91 | 0.05 | 0.00769997 | 1.85E-01 | blue |
| ENSG00000196843 | ARID5A | 0.15 | 1.11 | 0.06 | 0.00770208 | 1.85E-01 | brown4 |
| ENSG00000138604 | GLCE | -0.10 | 0.93 | 0.04 | 0.00775332 | 1.86E-01 | brown |
| ENSG00000083444 | PLOD1 | 0.11 | 1.08 | 0.04 | 0.00779463 | 1.86E-01 | brown4 |
| ENSG00000120820 | GLT8D2 | -0.10 | 0.93 | 0.04 | 0.00779747 | 1.86E-01 | lightsteelblue1 |
| ENSG00000109046 | WSB1 | -0.10 | 0.93 | 0.04 | 0.00786987 | 1.87E-01 | blue |
| ENSG00000163531 | NFASC | 0.11 | 1.08 | 0.04 | 0.00788069 | 1.87E-01 | blue |
| ENSG00000115170 | ACVR1 | -0.09 | 0.94 | 0.03 | 0.00788568 | 1.87E-01 | brown |
| ENSG00000186081 | KRT5 | -0.16 | 0.90 | 0.06 | 0.00790178 | 1.87E-01 | darkolivegreen |
| ENSG00000133216 | EPHB2 | -0.09 | 0.94 | 0.03 | 0.00790614 | 1.87E-01 | grey60 |
| ENSG00000102595 | UGGT2 | -0.07 | 0.95 | 0.03 | 0.00790913 | 1.87E-01 | darkgrey |
| ENSG00000150275 | PCDH15 | -0.11 | 0.93 | 0.04 | 0.00791685 | 1.87E-01 | paleturquoise |
| ENSG00000111247 | RAD51AP1 | -0.12 | 0.92 | 0.05 | 0.00792547 | 1.87E-01 | darkgrey |
| ENSG00000150938 | CRIM1 | 0.08 | 1.06 | 0.03 | 0.00797796 | 1.88E-01 | brown4 |
| ENSG00000269067 | ZNF728 | 0.15 | 1.11 | 0.06 | 0.00798248 | 1.88E-01 | brown4 |
| ENSG00000185053 | SGCZ | -0.11 | 0.93 | 0.04 | 0.00801883 | 1.88E-01 | brown |
| ENSG00000184260 | HIST2H2AC | 0.14 | 1.10 | 0.05 | 0.00802312 | 1.88E-01 | black |
| ENSG00000166887 | VPS39 | 0.06 | 1.04 | 0.02 | 0.00803357 | 1.88E-01 | darkolivegreen |
| ENSG00000123243 | ITIH5 | -0.15 | 0.90 | 0.06 | 0.0080343 | 1.88E-01 | lightgreen |
| ENSG00000122035 | RASL11A | -0.15 | 0.90 | 0.05 | 0.00807046 | 1.88E-01 | darkolivegreen |
| ENSG00000124440 | HIF3A | 0.15 | 1.11 | 0.06 | 0.00807755 | 1.88E-01 | black |
| ENSG00000198914 | POU3F3 | -0.09 | 0.94 | 0.03 | 0.00808053 | 1.88E-01 | paleturquoise |
| ENSG00000157045 | NTAN1 | -0.08 | 0.94 | 0.03 | 0.00815256 | 1.89E-01 | brown |
| ENSG00000120451 | SNX19 | 0.06 | 1.04 | 0.02 | 0.00821642 | 1.90E-01 | blue |
| ENSG00000147394 | ZNF185 | 0.11 | 1.08 | 0.04 | 0.00822465 | 1.90E-01 | blue |
| ENSG00000170962 | PDGFD | -0.14 | 0.90 | 0.05 | 0.00824689 | 1.90E-01 | thistle2 |
| ENSG00000230797 | YY2 | 0.14 | 1.10 | 0.05 | 0.00825994 | 1.90E-01 | paleturquoise |
| ENSG00000143603 | KCNN3 | 0.14 | 1.10 | 0.05 | 0.00827072 | 1.90E-01 | black |
| ENSG00000103460 | TOX3 | -0.13 | 0.92 | 0.05 | 0.00832075 | 1.91E-01 | thistle2 |
| ENSG00000167676 | PLIN4 | 0.15 | 1.11 | 0.06 | 0.00834727 | 1.91E-01 | brown4 |
| ENSG00000155366 | RHOC | 0.14 | 1.10 | 0.05 | 0.00835267 | 1.91E-01 | brown4 |
| ENSG00000179361 | ARID3B | -0.11 | 0.92 | 0.04 | 0.00835816 | 1.91E-01 | yellow |
| ENSG00000078237 | C12orf5 | -0.10 | 0.93 | 0.04 | 0.008359 | 1.91E-01 | brown |
| ENSG00000174989 | FBXW8 | -0.08 | 0.95 | 0.03 | 0.00838123 | 1.91E-01 | yellow |
| ENSG00000172987 | HPSE2 | -0.15 | 0.90 | 0.06 | 0.00841038 | 1.91E-01 | salmon4 |
| ENSG00000120159 | CAAP1 | -0.08 | 0.95 | 0.03 | 0.00841363 | 1.91E-01 | darkgrey |
| ENSG00000262576 | PCDHGA4 | 0.11 | 1.08 | 0.04 | 0.00842448 | 1.91E-01 | brown4 |
| ENSG00000035115 | SH3YL1 | -0.07 | 0.95 | 0.03 | 0.00848065 | 1.92E-01 | turquoise |
| ENSG00000071205 | ARHGAP10 | -0.12 | 0.92 | 0.05 | 0.00853569 | 1.93E-01 | mediumpurple3 |
| ENSG00000244405 | ETV5 | -0.14 | 0.91 | 0.05 | 0.0085653 | 1.93E-01 | darkslateblue |
| ENSG00000163116 | STPG2 | -0.13 | 0.92 | 0.05 | 0.00864464 | 1.95E-01 | paleturquoise |
| ENSG00000163702 | IL17RC | 0.11 | 1.08 | 0.04 | 0.00875604 | 1.97E-01 | brown4 |
| ENSG00000171291 | ZNF439 | -0.07 | 0.95 | 0.03 | 0.00877434 | 1.97E-01 | paleturquoise |
| ENSG00000140265 | ZSCAN29 | -0.09 | 0.94 | 0.03 | 0.00879044 | 1.97E-01 | blue |
| ENSG00000087269 | NOP14 | 0.07 | 1.05 | 0.03 | 0.00879594 | 1.97E-01 | darkolivegreen |
| ENSG00000136877 | FPGS | 0.09 | 1.06 | 0.03 | 0.00887958 | 1.99E-01 | darkolivegreen |

| | | | | | | | |
|---|---|---|---|---|---|---|---|
| ENSG00000137285 | TUBB2B | 0.15 | 1.11 | 0.06 | 0.0089201 | 1.99E-01 | brown4 |
| ENSG00000139899 | CBLN3 | -0.15 | 0.90 | 0.06 | 0.00895549 | 2.00E-01 | grey |
| ENSG00000189060 | H1F0 | 0.11 | 1.08 | 0.04 | 0.00904759 | 2.01E-01 | grey60 |
| ENSG00000163788 | SNRK | -0.08 | 0.95 | 0.03 | 0.00905232 | 2.01E-01 | brown |
| ENSG00000100815 | TRIP11 | -0.08 | 0.95 | 0.03 | 0.00914009 | 2.03E-01 | paleturquoise |
| ENSG00000187091 | PLCD1 | 0.13 | 1.09 | 0.05 | 0.00914883 | 2.03E-01 | brown4 |
| ENSG00000164691 | TAGAP | -0.11 | 0.93 | 0.04 | 0.00925569 | 2.05E-01 | turquoise |
| ENSG00000117298 | ECE1 | 0.10 | 1.07 | 0.04 | 0.0092937 | 2.05E-01 | brown4 |
| ENSG00000039319 | ZFYVE16 | -0.11 | 0.93 | 0.04 | 0.00930985 | 2.05E-01 | blue |
| ENSG00000254726 | MEX3A | 0.15 | 1.11 | 0.06 | 0.00932791 | 2.05E-01 | brown4 |
| ENSG00000112981 | NME5 | -0.11 | 0.93 | 0.04 | 0.0093395 | 2.05E-01 | lightsteelblue1 |
| ENSG00000131459 | GFPT2 | 0.12 | 1.09 | 0.05 | 0.00938545 | 2.06E-01 | plum2 |
| ENSG00000172469 | MANEA | -0.10 | 0.93 | 0.04 | 0.00942304 | 2.06E-01 | paleturquoise |
| ENSG00000151892 | GFRA1 | 0.13 | 1.09 | 0.05 | 0.0094268 | 2.06E-01 | black |
| ENSG00000167384 | ZNF180 | -0.09 | 0.94 | 0.03 | 0.00942787 | 2.06E-01 | brown |
| ENSG00000152580 | IGSF10 | -0.12 | 0.92 | 0.05 | 0.00943042 | 2.06E-01 | mediumpurple3 |
| ENSG00000102287 | GABRE | 0.15 | 1.11 | 0.06 | 0.00944798 | 2.06E-01 | brown4 |
| ENSG00000167528 | ZNF641 | -0.08 | 0.95 | 0.03 | 0.00945588 | 2.06E-01 | brown |
| ENSG00000150551 | LYPD1 | -0.14 | 0.91 | 0.05 | 0.009483 | 2.06E-01 | salmon4 |
| ENSG00000139679 | LPAR6 | -0.12 | 0.92 | 0.05 | 0.009507 | 2.06E-01 | lightcyan |
| ENSG00000130766 | SESN2 | 0.11 | 1.08 | 0.04 | 0.00953718 | 2.06E-01 | yellow |
| ENSG00000171208 | NETO2 | -0.11 | 0.93 | 0.04 | 0.00955732 | 2.06E-01 | lightsteelblue1 |
| ENSG00000137731 | FXYD2 | 0.15 | 1.11 | 0.06 | 0.00956555 | 2.06E-01 | yellow |
| ENSG00000104897 | SF3A2 | 0.11 | 1.08 | 0.04 | 0.0095975 | 2.06E-01 | yellow |
| ENSG00000181026 | AEN | -0.12 | 0.92 | 0.05 | 0.0096039 | 2.06E-01 | brown4 |
| ENSG00000261934 | PCDHGA9 | 0.09 | 1.07 | 0.04 | 0.00961656 | 2.06E-01 | brown4 |
| ENSG00000005007 | UPF1 | 0.08 | 1.06 | 0.03 | 0.00962633 | 2.06E-01 | yellow |
| ENSG00000139163 | ETNK1 | 0.10 | 1.07 | 0.04 | 0.00963416 | 2.06E-01 | darkgrey |
| ENSG00000118690 | ARMC2 | -0.07 | 0.95 | 0.03 | 0.00964576 | 2.06E-01 | yellow |
| ENSG00000144730 | IL17RD | 0.13 | 1.09 | 0.05 | 0.0096618 | 2.06E-01 | black |
| ENSG00000011295 | TTC19 | 0.07 | 1.05 | 0.03 | 0.00969302 | 2.06E-01 | brown |
| ENSG00000197261 | C6orf141 | -0.15 | 0.90 | 0.06 | 0.00969422 | 2.06E-01 | mediumpurple3 |
| ENSG00000107077 | KDM4C | -0.06 | 0.96 | 0.02 | 0.00969539 | 2.06E-01 | paleturquoise |
| ENSG00000144452 | ABCA12 | -0.14 | 0.91 | 0.05 | 0.00971797 | 2.06E-01 | black |
| ENSG00000198832 | SELM | 0.11 | 1.08 | 0.04 | 0.00971845 | 2.06E-01 | darkolivegreen |
| ENSG00000063978 | RNF4 | 0.06 | 1.04 | 0.02 | 0.00975688 | 2.06E-01 | skyblue3 |
| ENSG00000176956 | LY6H | 0.12 | 1.08 | 0.05 | 0.00976124 | 2.06E-01 | darkolivegreen |
| ENSG00000189269 | C22orf43 | -0.15 | 0.90 | 0.06 | 0.00977553 | 2.06E-01 | yellow |
| ENSG00000235711 | ANKRD34C | -0.13 | 0.92 | 0.05 | 0.00977718 | 2.06E-01 | lightsteelblue1 |
| ENSG00000179933 | C14orf119 | -0.10 | 0.93 | 0.04 | 0.00978207 | 2.06E-01 | brown |
| ENSG00000113532 | ST8SIA4 | -0.11 | 0.93 | 0.04 | 0.00978417 | 2.06E-01 | brown |
| ENSG00000130643 | CALY | 0.11 | 1.08 | 0.04 | 0.00981415 | 2.06E-01 | darkolivegreen |
| ENSG00000163900 | TMEM41A | 0.09 | 1.06 | 0.03 | 0.00982094 | 2.06E-01 | darkgrey |
| ENSG00000138092 | CENPO | -0.08 | 0.95 | 0.03 | 0.00991985 | 2.08E-01 | grey60 |
| ENSG00000167178 | ISLR2 | 0.14 | 1.10 | 0.06 | 0.0099558 | 2.08E-01 | yellow |
| ENSG00000058668 | ATP2B4 | 0.08 | 1.06 | 0.03 | 0.00996589 | 2.08E-01 | darkorange2 |
| ENSG00000168710 | AHCYL1 | 0.12 | 1.09 | 0.05 | 0.00997302 | 2.08E-01 | black |
| ENSG00000122085 | MTERFD2 | -0.06 | 0.96 | 0.02 | 0.00998491 | 2.08E-01 | grey |
| ENSG00000163377 | FAM19A4 | -0.14 | 0.91 | 0.05 | 0.00999685 | 2.08E-01 | paleturquoise |
| ENSG00000159423 | ALDH4A1 | 0.13 | 1.10 | 0.05 | 0.01001237 | 2.08E-01 | black |

| | | | | | | | |
|---|---|---|---|---|---|---|---|
| ENSG00000169744 | LDB2 | -0.09 | 0.94 | 0.03 | 0.01003171 | 2.08E-01 | lightsteelblue1 |
| ENSG00000152518 | ZFP36L2 | -0.14 | 0.91 | 0.05 | 0.01004474 | 2.08E-01 | paleturquoise |
| ENSG00000258484 | SPESP1 | 0.15 | 1.11 | 0.06 | 0.01006303 | 2.08E-01 | navajowhite2 |
| ENSG00000140859 | KIFC3 | -0.09 | 0.94 | 0.03 | 0.01007607 | 2.08E-01 | darkolivegreen |
| ENSG00000170011 | MYRIP | 0.08 | 1.06 | 0.03 | 0.01008406 | 2.08E-01 | saddlebrown |
| ENSG00000115556 | PLCD4 | 0.13 | 1.10 | 0.05 | 0.01011039 | 2.08E-01 | brown4 |
| ENSG00000144152 | FBLN7 | -0.15 | 0.90 | 0.06 | 0.01014767 | 2.09E-01 | grey60 |
| ENSG00000075884 | ARHGAP15 | -0.14 | 0.91 | 0.05 | 0.01019401 | 2.09E-01 | lightcyan |
| ENSG00000104497 | SNX16 | -0.10 | 0.93 | 0.04 | 0.01020439 | 2.09E-01 | brown |
| ENSG00000069493 | CLEC2D | -0.12 | 0.92 | 0.05 | 0.01031098 | 2.11E-01 | paleturquoise |
| ENSG00000134115 | CNTN6 | -0.13 | 0.91 | 0.05 | 0.01031124 | 2.11E-01 | darkorange2 |
| ENSG00000169223 | LMAN2 | 0.09 | 1.06 | 0.03 | 0.01032501 | 2.11E-01 | darkolivegreen |
| ENSG00000105607 | GCDH | 0.10 | 1.07 | 0.04 | 0.01033129 | 2.11E-01 | brown4 |
| ENSG00000152457 | DCLRE1C | -0.07 | 0.95 | 0.03 | 0.010353 | 2.11E-01 | paleturquoise |
| ENSG00000157514 | TSC22D3 | 0.12 | 1.09 | 0.05 | 0.01036153 | 2.11E-01 | brown4 |
| ENSG00000185122 | HSF1 | 0.10 | 1.07 | 0.04 | 0.01044656 | 2.12E-01 | yellow |
| ENSG00000134602 | MST4 | -0.13 | 0.91 | 0.05 | 0.01046206 | 2.12E-01 | brown |
| ENSG00000088826 | SMOX | 0.12 | 1.09 | 0.05 | 0.01049956 | 2.13E-01 | brown4 |
| ENSG00000058262 | SEC61A1 | 0.09 | 1.06 | 0.03 | 0.01053578 | 2.13E-01 | brown4 |
| ENSG00000142233 | NTN5 | 0.14 | 1.10 | 0.06 | 0.01055072 | 2.13E-01 | yellow |
| ENSG00000105855 | ITGB8 | 0.13 | 1.10 | 0.05 | 0.01057221 | 2.13E-01 | paleturquoise |
| ENSG00000164930 | FZD6 | 0.12 | 1.08 | 0.05 | 0.01059811 | 2.13E-01 | brown4 |
| ENSG00000163939 | PBRM1 | -0.04 | 0.97 | 0.02 | 0.01061449 | 2.13E-01 | paleturquoise |
| ENSG00000147588 | PMP2 | -0.14 | 0.91 | 0.05 | 0.01063996 | 2.14E-01 | paleturquoise |
| ENSG00000166444 | ST5 | 0.08 | 1.06 | 0.03 | 0.01067262 | 2.14E-01 | yellow |
| ENSG00000152413 | HOMER1 | -0.11 | 0.92 | 0.04 | 0.01069987 | 2.14E-01 | brown |
| ENSG00000196872 | KIAA1211L | 0.10 | 1.07 | 0.04 | 0.01070032 | 2.14E-01 | yellow |
| ENSG00000179846 | NKPD1 | 0.13 | 1.09 | 0.05 | 0.01082142 | 2.16E-01 | yellow |
| ENSG00000112715 | VEGFA | -0.15 | 0.90 | 0.06 | 0.01082653 | 2.16E-01 | grey |
| ENSG00000157890 | MEGF11 | 0.10 | 1.07 | 0.04 | 0.01091383 | 2.17E-01 | brown4 |
| ENSG00000206140 | TMEM191C | -0.14 | 0.91 | 0.05 | 0.01094089 | 2.17E-01 | grey60 |
| ENSG00000184557 | SOCS3 | 0.11 | 1.08 | 0.04 | 0.01094095 | 2.17E-01 | brown4 |
| ENSG00000184601 | C14orf180 | 0.15 | 1.11 | 0.06 | 0.01096182 | 2.17E-01 | yellow |
| ENSG00000099998 | GGT5 | 0.15 | 1.11 | 0.06 | 0.01097615 | 2.17E-01 | brown4 |
| ENSG00000154813 | DPH3 | -0.09 | 0.94 | 0.04 | 0.01097644 | 2.17E-01 | brown |
| ENSG00000122970 | IFT81 | -0.07 | 0.95 | 0.03 | 0.01098579 | 2.17E-01 | darkgrey |
| ENSG00000153012 | LGI2 | -0.13 | 0.92 | 0.05 | 0.01101418 | 2.17E-01 | thistle2 |
| ENSG00000175471 | MCTP1 | 0.08 | 1.06 | 0.03 | 0.01106783 | 2.18E-01 | brown |
| ENSG00000139910 | NOVA1 | -0.07 | 0.95 | 0.03 | 0.01111462 | 2.19E-01 | lightsteelblue1 |
| ENSG00000137509 | PRCP | 0.10 | 1.07 | 0.04 | 0.01112968 | 2.19E-01 | brown4 |
| ENSG00000099715 | PCDH11Y | -0.14 | 0.91 | 0.06 | 0.01124188 | 2.20E-01 | navajowhite2 |
| ENSG00000196369 | SRGAP2B | -0.08 | 0.94 | 0.03 | 0.0113085 | 2.21E-01 | blue |
| ENSG00000159885 | ZNF222 | -0.10 | 0.93 | 0.04 | 0.0113578 | 2.22E-01 | brown |
| ENSG00000076242 | MLH1 | -0.06 | 0.96 | 0.02 | 0.01138662 | 2.22E-01 | paleturquoise |
| ENSG00000140332 | TLE3 | 0.11 | 1.08 | 0.04 | 0.01139903 | 2.22E-01 | yellow |
| ENSG00000152492 | CCDC50 | -0.09 | 0.94 | 0.04 | 0.01139999 | 2.22E-01 | paleturquoise |
| ENSG00000204161 | C10orf128 | -0.15 | 0.90 | 0.06 | 0.01141149 | 2.22E-01 | blue |
| ENSG00000233436 | BTBD18 | 0.11 | 1.08 | 0.05 | 0.01144839 | 2.22E-01 | yellow |
| ENSG00000138646 | HERC5 | 0.14 | 1.10 | 0.06 | 0.01149426 | 2.23E-01 | palevioletred3 |
| ENSG00000183624 | HMCES | 0.07 | 1.05 | 0.03 | 0.01152181 | 2.23E-01 | saddlebrown |

| | | | | | | | |
|---|---|---|---|---|---|---|---|
| ENSG00000153391 | INO80C | -0.08 | 0.94 | 0.03 | 0.01158889 | 2.24E-01 | yellow |
| ENSG00000168079 | SCARA5 | -0.15 | 0.90 | 0.06 | 0.01161566 | 2.24E-01 | lightgreen |
| ENSG00000153446 | C16orf89 | 0.14 | 1.11 | 0.06 | 0.01163451 | 2.24E-01 | brown4 |
| ENSG00000149292 | TTC12 | 0.13 | 1.09 | 0.05 | 0.01163634 | 2.24E-01 | black |
| ENSG00000149346 | SLX4IP | -0.09 | 0.94 | 0.04 | 0.01164351 | 2.24E-01 | paleturquoise |
| ENSG00000164600 | NEUROD6 | -0.14 | 0.91 | 0.06 | 0.01164895 | 2.24E-01 | brown |
| ENSG00000197776 | KLHDC1 | -0.08 | 0.94 | 0.03 | 0.01173995 | 2.25E-01 | paleturquoise |
| ENSG00000150756 | FAM173B | -0.09 | 0.94 | 0.04 | 0.01174329 | 2.25E-01 | paleturquoise |
| ENSG00000128815 | WDFY4 | -0.14 | 0.90 | 0.06 | 0.0117506 | 2.25E-01 | lightcyan |
| ENSG00000171885 | AQP4 | 0.15 | 1.11 | 0.06 | 0.01185856 | 2.27E-01 | black |
| ENSG00000176204 | LRRTM4 | -0.09 | 0.94 | 0.04 | 0.01187007 | 2.27E-01 | mediumpurple3 |
| ENSG00000148123 | LPPR1 | -0.14 | 0.91 | 0.05 | 0.01196361 | 2.28E-01 | blue |
| ENSG00000151715 | TMEM45B | -0.13 | 0.92 | 0.05 | 0.01198614 | 2.28E-01 | grey |
| ENSG00000196867 | ZFP28 | -0.06 | 0.96 | 0.03 | 0.01202836 | 2.29E-01 | brown |
| ENSG00000197375 | SLC22A5 | 0.09 | 1.07 | 0.04 | 0.01206882 | 2.29E-01 | yellow |
| ENSG00000188730 | VWC2 | -0.12 | 0.92 | 0.05 | 0.01213177 | 2.30E-01 | thistle2 |
| ENSG00000188070 | C11orf95 | 0.08 | 1.06 | 0.03 | 0.0121656 | 2.30E-01 | brown |
| ENSG00000164591 | MYOZ3 | -0.12 | 0.92 | 0.05 | 0.01217091 | 2.30E-01 | darkolivegreen |
| ENSG00000145861 | C1QTNF2 | -0.12 | 0.92 | 0.05 | 0.01218166 | 2.30E-01 | grey60 |
| ENSG00000198003 | CCDC151 | -0.13 | 0.92 | 0.05 | 0.01230856 | 2.32E-01 | grey60 |
| ENSG00000159445 | THEM4 | -0.08 | 0.95 | 0.03 | 0.01236853 | 2.33E-01 | grey |
| ENSG00000184988 | TMEM106A | -0.13 | 0.91 | 0.05 | 0.01237096 | 2.33E-01 | lightcyan |
| ENSG00000133121 | STARD13 | 0.09 | 1.06 | 0.04 | 0.01239155 | 2.33E-01 | blue |
| ENSG00000174600 | CMKLR1 | -0.14 | 0.91 | 0.06 | 0.01240695 | 2.33E-01 | lightcyan |
| ENSG00000128805 | ARHGAP22 | -0.13 | 0.91 | 0.05 | 0.01247137 | 2.34E-01 | blue |
| ENSG00000157916 | RER1 | 0.06 | 1.04 | 0.02 | 0.01248318 | 2.34E-01 | blue |
| ENSG00000175764 | TTLL11 | -0.09 | 0.94 | 0.04 | 0.012512 | 2.34E-01 | blue |
| ENSG00000123213 | NLN | -0.07 | 0.95 | 0.03 | 0.01254691 | 2.34E-01 | brown |
| ENSG00000155636 | RBM45 | -0.07 | 0.95 | 0.03 | 0.01257413 | 2.34E-01 | paleturquoise |
| ENSG00000183255 | PTTG1IP | 0.13 | 1.09 | 0.05 | 0.01257466 | 2.34E-01 | brown4 |
| ENSG00000111652 | COPS7A | -0.09 | 0.94 | 0.03 | 0.01259253 | 2.34E-01 | darkolivegreen |
| ENSG00000221986 | MYBPHL | 0.14 | 1.10 | 0.06 | 0.0126014 | 2.34E-01 | brown4 |
| ENSG00000077235 | GTF3C1 | 0.06 | 1.04 | 0.02 | 0.01261292 | 2.34E-01 | grey60 |
| ENSG00000056736 | IL17RB | 0.12 | 1.09 | 0.05 | 0.01269121 | 2.35E-01 | black |
| ENSG00000056972 | TRAF3IP2 | 0.10 | 1.08 | 0.04 | 0.0127003 | 2.35E-01 | brown4 |
| ENSG00000198771 | RCSD1 | -0.12 | 0.92 | 0.05 | 0.0127521 | 2.36E-01 | lightcyan |
| ENSG00000232119 | MCTS1 | -0.08 | 0.94 | 0.03 | 0.01284456 | 2.37E-01 | brown |
| ENSG00000173198 | CYSLTR1 | -0.14 | 0.91 | 0.06 | 0.01290041 | 2.38E-01 | lightcyan |
| ENSG00000101193 | GID8 | 0.05 | 1.03 | 0.02 | 0.01291132 | 2.38E-01 | brown |
| ENSG00000213903 | LTB4R | 0.14 | 1.10 | 0.05 | 0.0129489 | 2.38E-01 | yellow |
| ENSG00000163288 | GABRB1 | 0.08 | 1.06 | 0.03 | 0.01296746 | 2.38E-01 | black |
| ENSG00000148429 | USP6NL | -0.07 | 0.95 | 0.03 | 0.01300541 | 2.39E-01 | paleturquoise |
| ENSG00000101871 | MID1 | 0.12 | 1.09 | 0.05 | 0.01302206 | 2.39E-01 | black |
| ENSG00000183722 | LHFP | 0.11 | 1.08 | 0.04 | 0.01304311 | 2.39E-01 | brown4 |
| ENSG00000170417 | TMEM182 | -0.12 | 0.92 | 0.05 | 0.01308096 | 2.39E-01 | lightsteelblue1 |
| ENSG00000178573 | MAF | -0.11 | 0.92 | 0.05 | 0.01318228 | 2.40E-01 | brown |
| ENSG00000233932 | CTXN2 | -0.12 | 0.92 | 0.05 | 0.01318565 | 2.40E-01 | lightsteelblue1 |
| ENSG00000181523 | SGSH | 0.10 | 1.08 | 0.04 | 0.01326519 | 2.41E-01 | yellow |
| ENSG00000076555 | ACACB | 0.13 | 1.10 | 0.05 | 0.01326603 | 2.41E-01 | black |
| ENSG00000163884 | KLF15 | 0.13 | 1.10 | 0.05 | 0.01329287 | 2.41E-01 | brown4 |

| | | | | | | | |
|---|---|---|---|---|---|---|---|
| ENSG00000175344 | CHRNA7 | -0.13 | 0.91 | 0.05 | 0.01334687 | 2.42E-01 | thistle2 |
| ENSG00000138185 | ENTPD1 | -0.07 | 0.95 | 0.03 | 0.01337397 | 2.42E-01 | paleturquoise |
| ENSG00000187079 | TEAD1 | 0.10 | 1.07 | 0.04 | 0.01337623 | 2.42E-01 | black |
| ENSG00000138074 | SLC5A6 | -0.12 | 0.92 | 0.05 | 0.01339205 | 2.42E-01 | darkolivegreen |
| ENSG00000188486 | H2AFX | 0.10 | 1.07 | 0.04 | 0.01343747 | 2.43E-01 | yellow |
| ENSG00000182551 | ADI1 | 0.10 | 1.07 | 0.04 | 0.01349617 | 2.43E-01 | black |
| ENSG00000133878 | DUSP26 | 0.08 | 1.06 | 0.03 | 0.01350653 | 2.43E-01 | darkolivegreen |
| ENSG00000186591 | UBE2H | 0.06 | 1.04 | 0.02 | 0.0135286 | 2.43E-01 | brown4 |
| ENSG00000075240 | GRAMD4 | -0.08 | 0.95 | 0.03 | 0.01354124 | 2.43E-01 | grey60 |
| ENSG00000102878 | HSF4 | 0.11 | 1.08 | 0.05 | 0.01357758 | 2.44E-01 | tan |
| ENSG00000197372 | ZNF675 | -0.08 | 0.95 | 0.03 | 0.01365746 | 2.44E-01 | brown |
| ENSG00000086544 | ITPKC | 0.13 | 1.10 | 0.05 | 0.01367243 | 2.44E-01 | brown4 |
| ENSG00000185973 | TMLHE | -0.08 | 0.94 | 0.03 | 0.01368991 | 2.44E-01 | paleturquoise |
| ENSG00000149480 | MTA2 | 0.11 | 1.08 | 0.05 | 0.01370395 | 2.44E-01 | brown4 |
| ENSG00000155085 | AK9 | -0.08 | 0.95 | 0.03 | 0.01371475 | 2.44E-01 | paleturquoise |
| ENSG00000145700 | ANKRD31 | -0.12 | 0.92 | 0.05 | 0.01371854 | 2.44E-01 | blue |
| ENSG00000058335 | RASGRF1 | -0.11 | 0.93 | 0.04 | 0.01375805 | 2.45E-01 | grey60 |
| ENSG00000166596 | WDR16 | -0.11 | 0.93 | 0.04 | 0.01387111 | 2.46E-01 | lightsteelblue1 |
| ENSG00000102678 | FGF9 | -0.09 | 0.94 | 0.04 | 0.01387644 | 2.46E-01 | saddlebrown |
| ENSG00000181938 | GINS3 | -0.11 | 0.92 | 0.05 | 0.01391095 | 2.46E-01 | blue |
| ENSG00000168724 | DNAJC21 | -0.07 | 0.95 | 0.03 | 0.01392879 | 2.46E-01 | brown |
| ENSG00000165912 | PACSIN3 | -0.13 | 0.91 | 0.05 | 0.01399602 | 2.47E-01 | blue |
| ENSG00000138792 | ENPEP | 0.14 | 1.10 | 0.06 | 0.01400196 | 2.47E-01 | darkslateblue |
| ENSG00000188739 | RBM34 | 0.10 | 1.07 | 0.04 | 0.01401802 | 2.47E-01 | yellow |
| ENSG00000179476 | C14orf28 | -0.09 | 0.94 | 0.04 | 0.01403361 | 2.47E-01 | paleturquoise |
| ENSG00000137275 | RIPK1 | 0.09 | 1.06 | 0.04 | 0.01404179 | 2.47E-01 | brown4 |
| ENSG00000188677 | PARVB | 0.08 | 1.06 | 0.03 | 0.0140672 | 2.47E-01 | darkolivegreen |
| ENSG00000258839 | MC1R | 0.13 | 1.10 | 0.05 | 0.01410173 | 2.47E-01 | yellow |
| ENSG00000143669 | LYST | -0.06 | 0.96 | 0.02 | 0.01417656 | 2.48E-01 | lightsteelblue1 |

ID: Ensembl id of the gene
log2Fold change: log2 value of the fold change calculated using DeSeq2
FC: Fold change
pvalue: P value for the fold change of expression among AD and controls
padj: FDR adjusted pvalue
ModuleColor: The group assigned to each gene on the basis of co-expression by WGCNA
