# Supplementary material for: Analysis of whole genome-transcriptomic organization in brain to identify genes associated with alcoholism
Source: Transl Psychiatry. 2019 Feb 14;9:89. doi: 10.1038/s41398-019-0384-y (PMC6376002; doi:10.1038/s41398-019-0384-y)
Supplement: Supplementary file 3 — Supplementary Table 2 [file 41398_2019_384_MOESM3_ESM.pdf]

Supp table 2a: Differntialy expressed hub genes of Thistle2 module in brain of AD subjects

id	gene	log2FoldChange	lfcSE	pvalue	Mouse/ Rat brain (Diff expression P)	Region	Ethanol/ Treatment	Refernce
ENSG00000171189	GRIK1	-0.16	0.04	1.59E-04	3.00E-02	Frontal-cortex	AP vs nAP	Ferguson et al, 2017
ENSG00000189056	RELN	-0.20	0.06	5.04E-04	6.80E-03	whole brain	AP vs nAP	Kimpel et al, 2007
ENSG00000231764	DLX6-AS1	-0.18	0.06	1.18E-03				
ENSG00000146469	VIP	-0.16	0.06	4.16E-03	9.00E-04	Whole brain	AP vs nAP	Mulligan et al, 2006
ENSG00000157404	KIT	-0.14	0.05	5.77E-03	4.00E-02	Central Amygdala	AP vs nAP	Ferguson et al, 2017
ENSG00000017427	IGF1	-0.12	0.05	1.91E-02				
ENSG00000135824	RGS8	-0.13	0.06	2.31E-02				
ENSG00000172137	CALB2	-0.12	0.06	3.54E-02	1.90E-02	Frontal cortex	DID Drinking	Osterndorff-Kahanek et al, 2013
ENSG00000178568	ERBB4	-0.06	0.04	9.32E-02				
ENSG00000144355	DLX1	-0.08	0.05	1.32E-01	4.90E-03	whole brain	AP vs nAP	Mulligan et al, 2006
ENSG00000151136	BTBD11	-0.06	0.05	1.93E-01	2.00E-02	Frontal-cortex	Every other day drinking	Osterndorff-Kahanek et al, 2013

ID: Ensembl id of the gene
log2Fold change: log2 value of the fold change calculated using DeSeq2
Column F: Diffential expression P value in mouse or rat models
Column G: Region of the mouse/ rat brain used in the study
Column H: Description of the mouse model (e.g. Alcohol prefernce vs non prefernce; DID (Drinking in the dark)
Reference: Reference of the study

Supp table 2b: Differntialy expressed hub genes of Brown 4 module in brain of AD subjects

id	gene	log2FoldChar	lfcSE	pvalue	**Mouse/ Rat brain (Diff expression P)**	**Region**	**Ethanol/ Treatment**	**Refernce**
ENSG00000142089	IFITM3	0.27	0.06	4.52E-06	7.70E-04	Frontal-cortex	AP vs nAP	Osterndorff-Kahanek et al, 2013
ENSG00000147872	PLIN2	0.24	0.06	3.82E-05				
ENSG00000144908	ALDH1L1	0.22	0.06	1.52E-04	1.90E-02	Frontal-cortex	Chronic drinking	Osterndorff-Kahanek et al, 2013
ENSG00000151929	BAG3	0.21	0.06	1.60E-04				
ENSG00000148498	PARD3	0.17	0.05	1.21E-03				
ENSG00000185201	IFITM2	0.17	0.06	1.90E-03	1.20E-03	Frontal-cortex	AP vs nAP	Osterndorff-Kahanek et al, 2013
ENSG00000072952	MRVI1	0.17	0.06	2.30E-03				
ENSG00000067182	TNFRSF1A	0.16	0.06	5.20E-03				
ENSG00000077238	IL4R	0.16	0.06	6.06E-03				
ENSG00000051620	HEBP2	0.13	0.05	6.18E-03				
ENSG00000109113	RAB34	0.15	0.06	6.26E-03	7.20E-03	Frontal-cortex	AP vs nAP	Osterndorff-Kahanek et al, 2013
ENSG00000168309	FAM107A	0.15	0.05	6.71E-03	1.60E-03	Whole Brain	AP vs nAP	Mulligan et al, 2006
ENSG00000145623	OSMR	0.15	0.06	6.76E-03	7.70E-03	Whole Brain	AP vs nAP	Mulligan et al, 2006
ENSG00000197324	LRP10	0.14	0.05	7.17E-03				
ENSG00000155366	RHOC	0.14	0.05	8.35E-03	4.50E-05	Whole Brain	AP vs nAP	Mulligan et al, 2006
ENSG00000187091	PLCD1	0.13	0.05	9.15E-03				
ENSG00000183255	PTTG1IP	0.13	0.05	1.26E-02	4.30E-04	Frontal-cortex	AP vs nAP	Osterndorff-Kahanek et al, 2013
ENSG00000117519	CNN3	0.13	0.06	1.75E-02	1.20E-02	Whole Brain	AP vs nAP	Kimpel et al, 2007
ENSG00000182541	LIMK2	0.12	0.05	2.03E-02				
ENSG00000158710	TAGLN2	0.13	0.06	2.35E-02	2.80E-02	Frontal-cortex	AP vs nAP	Ferguson et al, 2017
ENSG00000148175	STOM	0.12	0.06	2.81E-02				
ENSG00000168610	STAT3	0.10	0.05	3.34E-02	7.00E-04	Whole Brain	AP vs nAP	Mulligan et al, 2006
ENSG00000135926	TMBIM1	0.12	0.06	3.75E-02	3.00E-03	Whole Brain	AP vs nAP	Mulligan et al, 2006
ENSG00000131981	LGALS3	0.11	0.06	6.71E-02				
ENSG00000129116	PALLD	0.08	0.05	9.48E-02				
ENSG00000198805	PNP	0.10	0.06	1.01E-01				
ENSG00000181826	RELL1	0.09	0.06	1.23E-01	4.20E-02	Frontal-cortex	DID drinking	Osterndorff-Kahanek et al, 2013
ENSG00000119408	NEK6	0.06	0.05	1.63E-01	1.20E-02	Whole Brain	AP vs nAP	Mulligan et al, 2006
ENSG00000137693	YAP1	0.07	0.05	1.73E-01				
ENSG00000031081	ARHGAP31	0.06	0.05	2.09E-01				
ENSG00000147065	MSN	0.07	0.05	2.22E-01	5.00E-03	Whole Brain	AP vs nAP	Mulligan et al, 2006
ENSG00000077150	NFKB2	0.07	0.06	2.27E-01				
ENSG00000162909	CAPN2	0.04	0.04	2.31E-01	1.10E-02	Frontal-cortex	AP vs nAP	Osterndorff-Kahanek et al, 2013
ENSG00000177119	ANO6	0.06	0.05	2.34E-01				
ENSG00000106565	TMEM176B	0.07	0.06	2.55E-01	2.30E-02	Frontal-cortex	AP vs nAP	Osterndorff-Kahanek et al, 2013
ENSG00000213719	CLIC1	0.06	0.06	2.66E-01				
ENSG00000168994	PXDC1	0.06	0.06	3.04E-01				
ENSG00000101608	MYL12A	0.04	0.05	4.93E-01				
ENSG00000124782	RREB1	0.02	0.05	6.95E-01	4.70E-02	Frontal-cortex	AP vs nAP	Osterndorff-Kahanek et al, 2013

ID: Ensembl id of the gene
log2Fold change: log2 value of the fold change calculated using DeSeq2
Column F: Diffential expression P value in mouse or rat models
Column G: Region of the mouse/ rat brain used in the study
Column H: Description of the mouse model (e.g. Alcohol prefernce vs non prefernce; DID (Drinking in the dark)
Reference: Reference of the study
