# Supplementary material for: Analysis of whole genome-transcriptomic organization in brain to identify genes associated with alcoholism
Source: Transl Psychiatry. 2019 Feb 14;9:89. doi: 10.1038/s41398-019-0384-y (PMC6376002; doi:10.1038/s41398-019-0384-y)
Supplement: Supplementary file 4 — Supplementary Figure 1 [file 41398_2019_384_MOESM4_ESM.pdf]

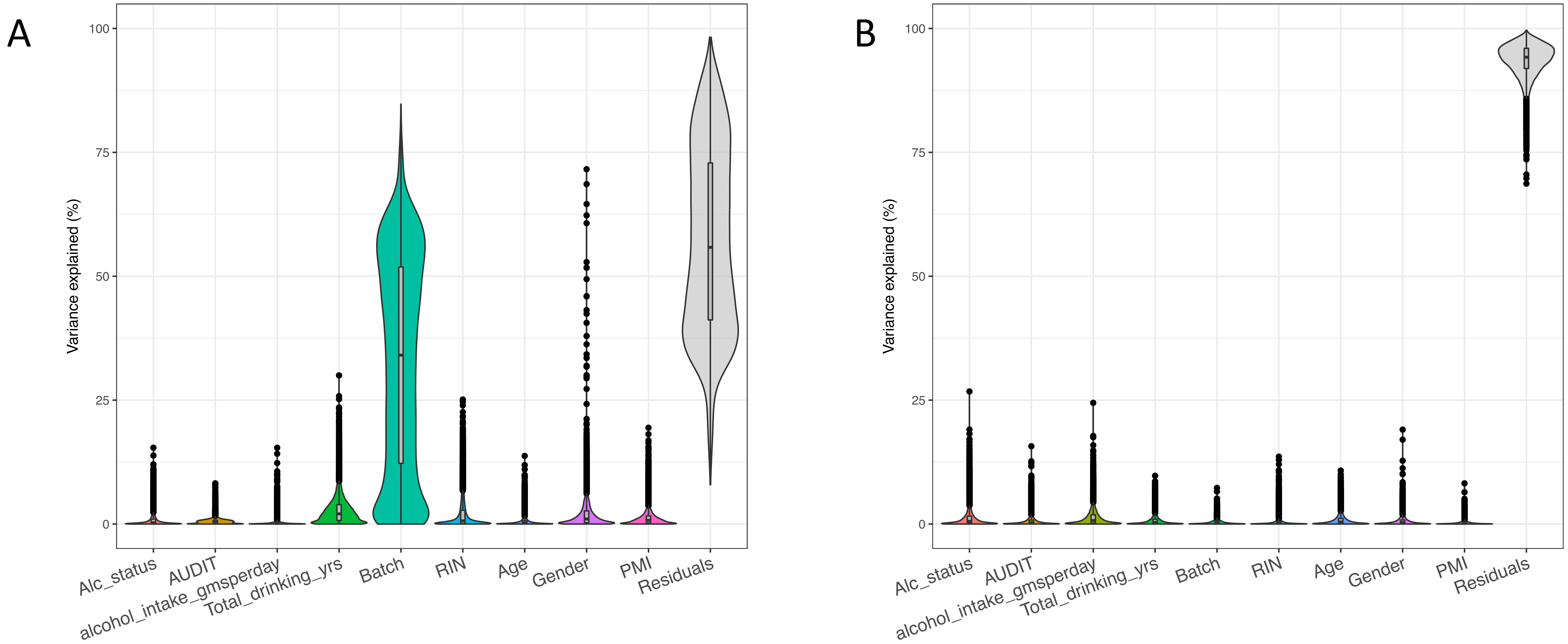

Supplementary figure 1: Violin plots depicting drivers of variation in gene expression. X-axis shows various traits/ confounders and y-axis shows the percentage of variance explained. Thickness of each violin bar depicts the proportion of genes corresponding to level of y-axis. (A) Sequencing batch was the major driver of variation in gene expression. (B) The variation partition plot after correction for batch, RIN, age, gender and PMI.
