# Supplementary material for: Analysis of whole genome-transcriptomic organization in brain to identify genes associated with alcoholism
Source: Transl Psychiatry. 2019 Feb 14;9:89. doi: 10.1038/s41398-019-0384-y (PMC6376002; doi:10.1038/s41398-019-0384-y)
Supplement: Supplementary file 5 — Supplementary Figure 2 [file 41398_2019_384_MOESM5_ESM.pdf]

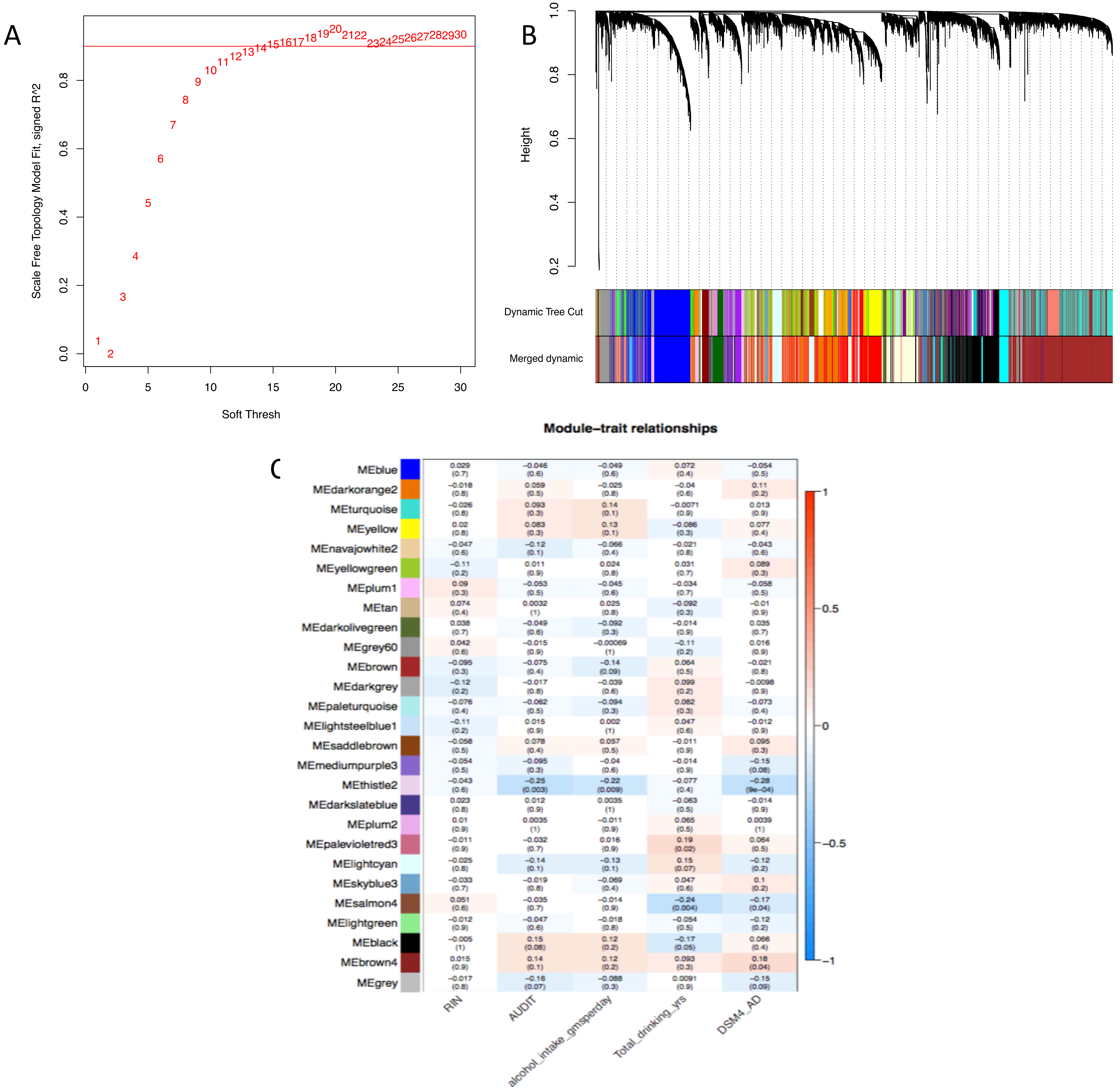

Supplementary Figure 2: **WGCNA derived modules of gene expression**. (A) Summary network indices (y-axes) as functions of the soft thresholding power (x-axes). Numbers in the plots indicate the corresponding soft thresholding powers. The plots indicate that approximate scale-free topology is attained around the soft-thresholding power of 14. (B) Gene dendrogram obtained by clustering the dissimilarity based on consensus Topological Overlap. The two color rows show the preliminary (unmerged) and the final, merged module assignments. (C) Correlation among module eigen values and alcohol related traits. Values in the parenthesis are the p values associated with correlation coeficient.
