# Supplementary figures and images for: Analysis of whole genome-transcriptomic organization in brain to identify genes associated with alcoholism
Source: Transl Psychiatry. 2019 Feb 14;9:89. doi: 10.1038/s41398-019-0384-y (PMC6376002; doi:10.1038/s41398-019-0384-y)

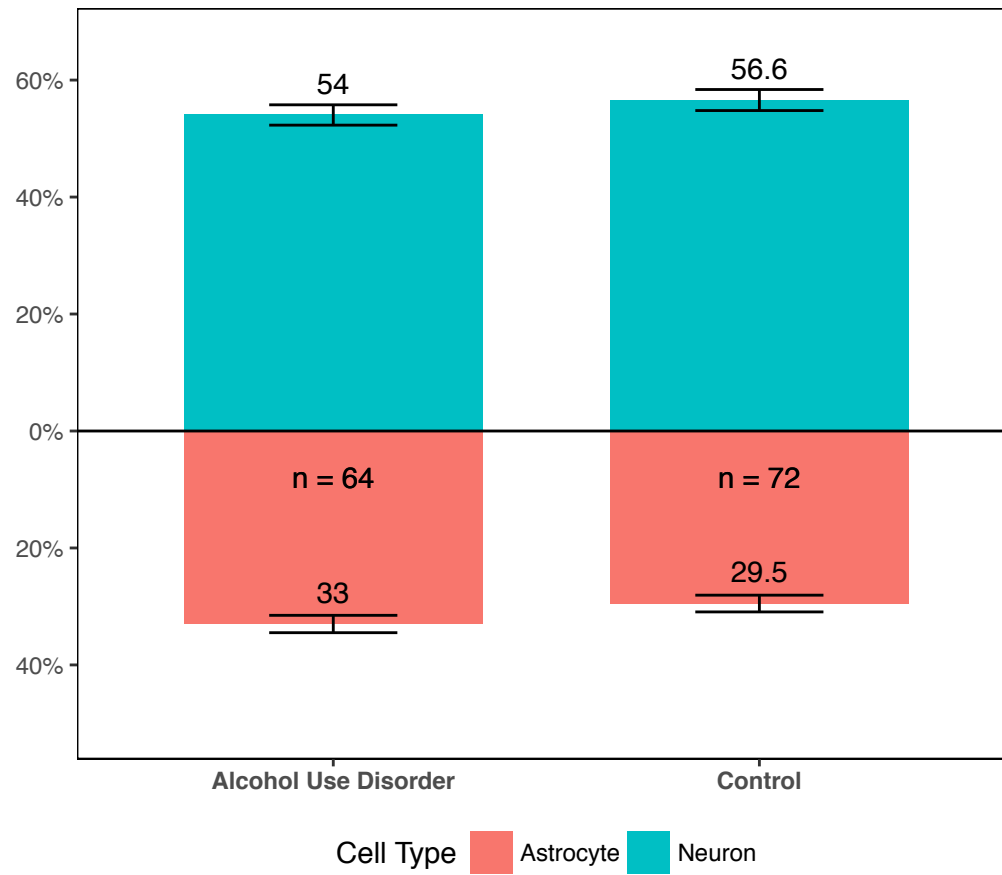

Supplementary Figure 3: Deconvolution of cell types in alcoholics and controls.

Supplement: Supplementary file 6 — Supplementary Figure 3 [file 41398_2019_384_MOESM6_ESM.pdf]

Supplementary  
Figure 4 (a)

**brown4 cor=0.21, p=8.1e-10**

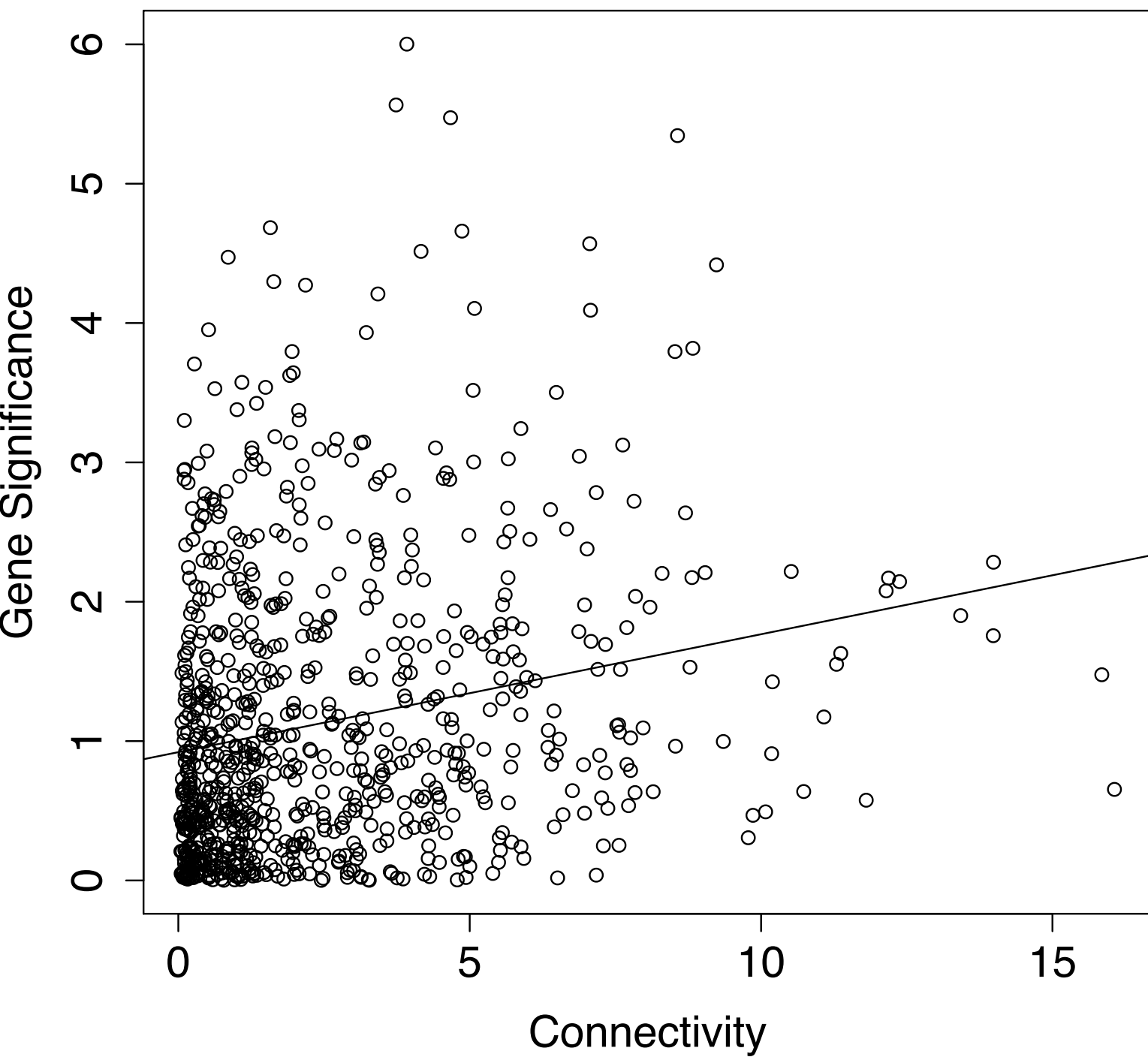

**thistle2 cor=0.013, p=0.92**

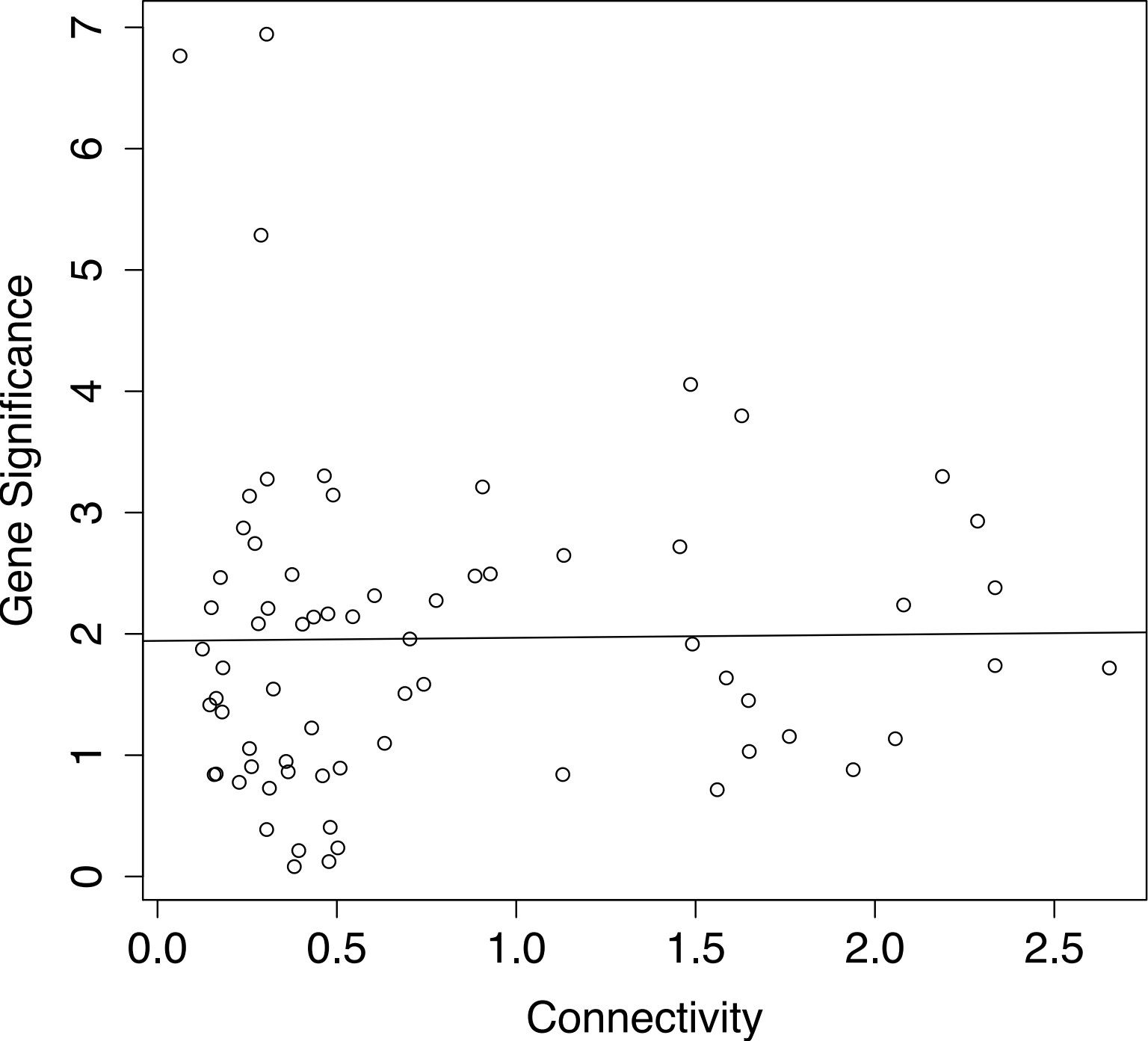

Supplement: Supplementary file 7 — Supplementary Figure 4 [file 41398_2019_384_MOESM7_ESM.pdf]
